# High-Efficiency Circularly Polarized Light-Emitting Diodes Based on Chiral Metal Nanoclusters

**DOI:** 10.1021/jacs.3c13065

**Published:** 2024-02-05

**Authors:** Jianxun Lu, Bingyao Shao, Ren-Wu Huang, Luis Gutiérrez-Arzaluz, Shulin Chen, Zhen Han, Jun Yin, Hongwei Zhu, Sergey Dayneko, Mohamed Nejib Hedhili, Xin Song, Peng Yuan, Chunwei Dong, Renqian Zhou, Makhsud I. Saidaminov, Shuang-Quan Zang, Omar F. Mohammed, Osman M. Bakr

**Affiliations:** † Division of Physical Science and Engineering, KAUST Catalysis Center (KCC), King Abdullah University of Science and Technology, Thuwal 23955-6900, Kingdom of Saudi Arabia; ‡ Key Laboratory of Crystalline Molecular Functional Materials, Henan International Joint Laboratory of Tumor Theranostical Cluster Materials, Green Catalysis Center, and College of Chemistry, Zhengzhou University, Zhengzhou 450001, China; § Division of Physical Science and Engineering, Advanced Membranes and Porous Materials Center (AMPM), King Abdullah University of Science and Technology, Thuwal 23955-6900, Kingdom of Saudi Arabia; ∥ Department of Applied Physics, The Hong Kong Polytechnic University, Hong Kong 999077, China; ⊥ Department of Electrical and Computer Engineering, University of Victoria, 3800 Finnerty Rd, Victoria, British Columbia, Canada V8P 5C2; # The Imaging and Characterization Core Lab, King Abdullah University of Science and Technology, Thuwal 23955-6900, Kingdom of Saudi Arabia

## Abstract

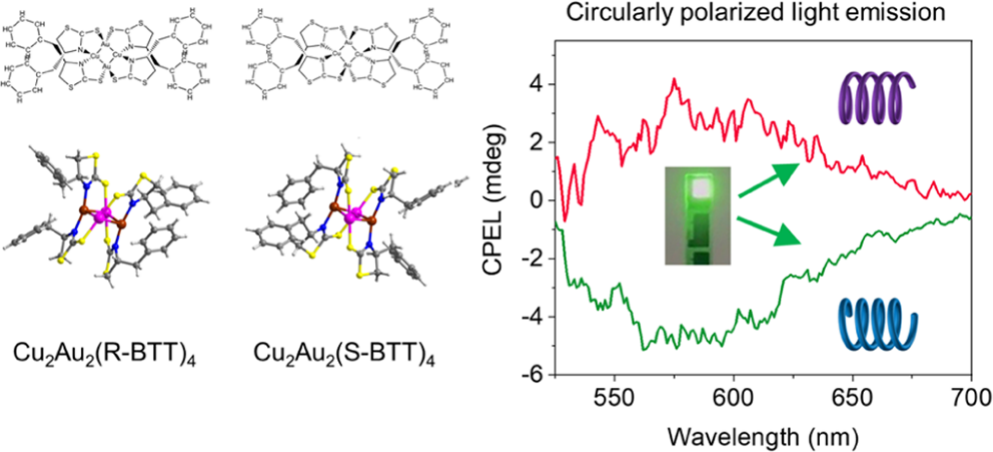

Circularly polarized light-emitting diodes (CP-LEDs) are critical for next-generation optical technologies, ranging from holography to quantum information processing. Currently deployed chiral luminescent materials, with their intricate synthesis and processing and limited efficiency, are the main bottleneck for CP-LEDs. Chiral metal nanoclusters (MNCs) are potential CP-LED materials, given their ease of synthesis and processability as well as diverse structures and excited states. However, their films are usually plagued by inferior electronic quality and aggregation-caused photoluminescence quenching, necessitating their incorporation into host materials; without such a scheme, MNC-based LEDs exhibit external quantum efficiencies (EQEs) < 10%. Herein, we achieve an efficiency leap for both CP-LEDs and cluster-based LEDs by using novel chiral MNCs with aggregation-induced emission enhancement. CP-LEDs using enantiopure MNC films attain EQEs of up to 23.5%. Furthermore, by incorporating host materials, the devices yield record EQEs of up to 36.5% for both CP-LEDs and cluster-based LEDs, along with electroluminescence dissymmetry factors (|*g*_EL_|) of around 1.0 × 10^–3^. These findings open a new avenue for advancing chiral light sources for next-generation optoelectronics.

## Introduction

Circularly polarized luminescence (CPL) has promising applications in three-dimensional holographic displays, augmented and virtual reality, optical data storage, biological imaging, and optical quantum information processing.^1−7^ Traditionally, circularly polarized light is obtained from unpolarized light through a linear polarizer and a quarter-wave plate. However, at least 50% of the photons will be lost in the process.^8,9^ In contrast, light-emitting diodes (LEDs) based on chiral luminescent materials, so-called circularly polarized light-emitting diodes (CP-LEDs), directly generate circularly polarized electroluminescence (CPEL), enabling simpler and efficient devices.^8,10,11^ Over the past few years, numerous chiral luminescent materials were introduced in the CP-LEDs, such as chiral lanthanide complexes,^12−14^ chiral small organic molecules,^15−17^ chiral conjected polymers,^18−20^ and chiral transition metal complexes.^21−23^ However, their intricate synthesis, complex processing, and limited efficiency have hindered the development of CP-LEDs.

Recently, chiral metal nanoclusters (MNCs) have emerged as attractive circularly polarized emitters, combining the advantages of organic and inorganic materials.^24,25^ MNCs are typically composed of several metal atoms, such as gold, silver, copper, and other transition metals, coordinated with organic ligands. The interaction between the metal atoms and ligands as well as the metal–metal synergistic effect plays a crucial role in determining the complex excited states and luminescence properties of these nanoclusters.^26^ By precisely tuning the number of metal atoms, ligand structures, and coordination strength, the excited states and luminescence properties may be tailored to suit specific applications.^27,28^

The efficiency of LEDs based on MNCs thus far has been limited by two primary issues. The presence of excessive ligands which leads to inferior electrical conductivity and electronic film quality adversely affects charge injection and transport within the LEDs.^29^ Moreover, when MNCs aggregate or come into proximity, their excited states can interact, resulting in nonradiative energy transfer and quenching of the luminescence.^30,31^ These factors require the integration of MNCs into host materials, which have shown promise in partially mitigating and ameliorating these issues, leading to an increase in the reported maximum external quantum efficiency (EQE) up to 29.4%.^32^ Nevertheless, in the majority of reported studies, the incorporation of at least 80 wt % host materials remains a prerequisite to ensure sufficient electrical conductivity of the emissive layer.^29,32,33^ On the other hand, the EQEs of LEDs based on pure MNCs, without host material integration, remain below 10%,^32,34−36^ underscoring the need to surmount MNC-LED challenges to harness their potential for high-efficiency optoelectronics.

Herein, we report high-efficiency CP-LEDs using novel chiral copper–gold MNCs protected by enantiopure R/S-4-benzylthiazolidine-2-thione (R/S-BTT) ligands. These enantiopure MNCs, namely Cu_2_Au_2_(R-BTT)_4_ and Cu_2_Au_2_(S-BTT)_4_, exhibit aggregation-induced emission enhancement (AIEE), endowing their solution-processed films with high photoluminescence quantum yields (ϕ_PL_) of up to ∼94 and 89%, respectively. CP-LEDs with pure MNC emissive layers achieved high EQEs of 23.5 and 20.8% for Cu_2_Au_2_(R-BTT)_4_ and Cu_2_Au_2_(S-BTT)_4_, respectively. Furthermore, by adding 20 wt % tris(4-carbazoyl-9-ylphenyl)amine (TCTA) into Cu_2_Au_2_(R-BTT)_4_, the devices displayed optimal energy band alignment and faster exciton transfer from singlet to triplet, yielding an EQE of 36.5%, highest reported to date for any CP-LEDs and cluster-based LEDs. Notably, these devices exhibited remarkable CPEL with a dissymmetry factor (|*g*_EL_|) of ∼1.0 × 10^–3^.

## Results and Discussion

### Optoelectronic Characteristics and Aggregation-Induced Emission Enhancement (AIEE) Behavior

**Figure 1:**
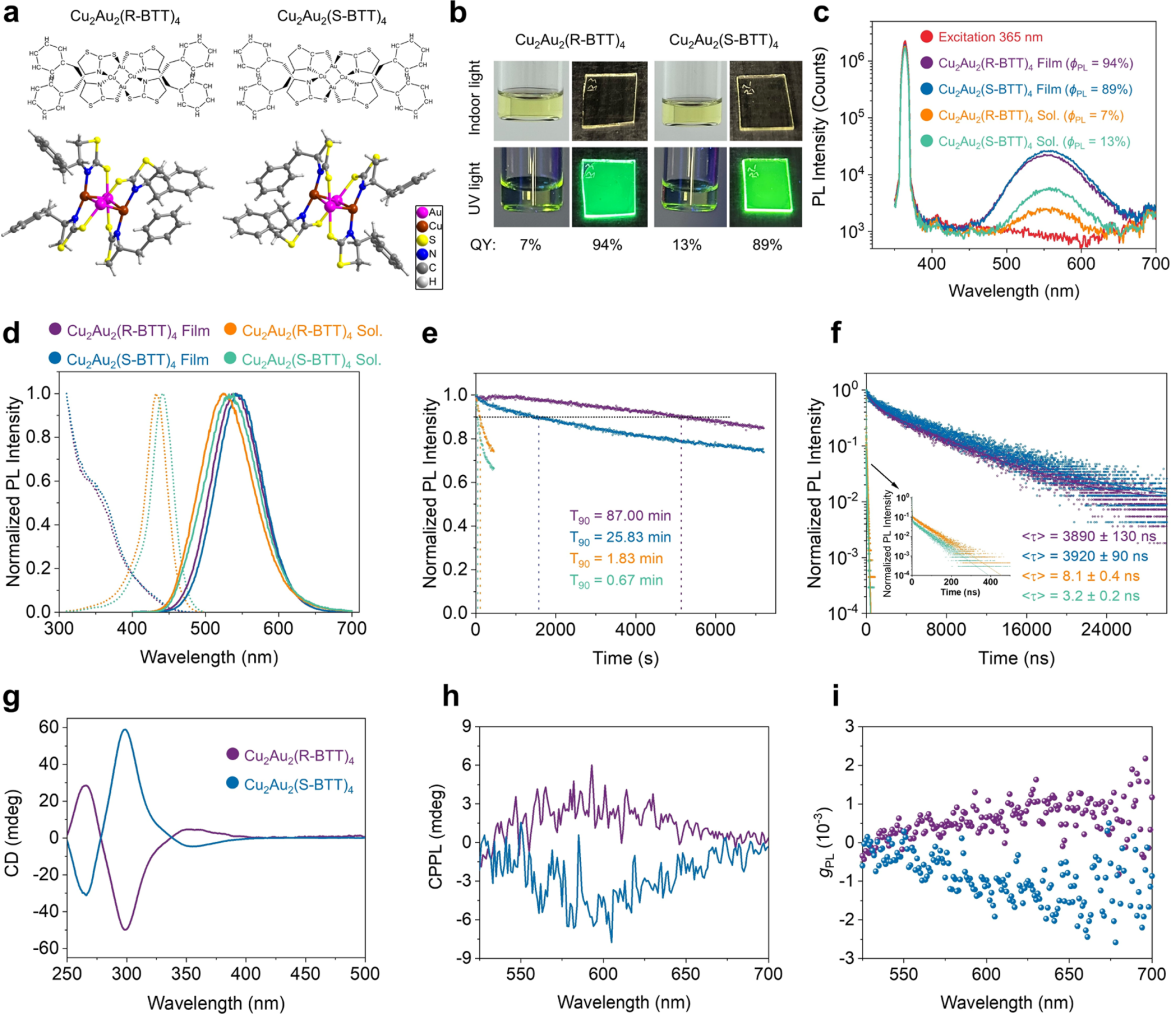
Structure, photoluminescence characteristics, and chirality of Cu_2_Au_2_(R-BTT)_4_ and Cu_2_Au_2_(S-BTT)_4_. (a) Molecular structure and ball–stick model of Cu_2_Au_2_(R/S-BTT)_4_. (b) Photographs under indoor light and UV light and (c) photoluminescence quantum efficiency spectra under an excitation at 365 nm of Cu_2_Au_2_(R/S-BTT)_4_ in solution and film states. (d–f) PL characteristic variation as Cu_2_Au_2_(R-BTT)_4_ and Cu_2_Au_2_(S-BTT)_4_ transfer from solutions to films. (d) Normalized PL emission (solid line) and normalized PL excitation (PLE, short dot) spectra. The PL and PLE spectra were excited and recorded at 365 and 540 nm, respectively. (e) PL intensity evolution curves under a constant excitation at 365 nm. The PL intensity was recorded at 540 nm. (f) TRPL decay curves. (g) CD spectra, (h) CPPL spectra, and (i) *g*_PL_ versus wavelength curves of Cu_2_Au_2_(R/S-BTT)_4_ films.

Cu_2_Au_2_(R-BTT)_4_ and Cu_2_Au_2_(S-BTT)_4_ are synthesized by a protocol for one-pot gram-scale production. Details of the synthesis processes are described in the Methods. The crystallographic data shown in Table S1 reveal that Cu_2_Au_2_(R-BTT)_4_ and Cu_2_Au_2_(S-BTT)_4_ crystallize in the chiral space group *P*2_1_ and their molecular and ball–stick structures are shown in Figure 1a. As shown in Figure S1, the crystallographic analysis reveals a distinctive planar tetranuclear structure composed of a Cu–Au alloy, protected by R/S-BTT ligands. The Cu–Au core displays quasi-rhombic geometry with two pairs of unequal opposite angles. The average Cu–Au distance of 2.847 Å is notably shorter than the sum of their van der Waals radii (3.06 Å), indicating the participation of metallophilic interactions in stabilizing the nanocluster structure. The ligands adopt a bidentate binding pattern, with nitrogen atoms bonding with the copper atoms and sulfur atoms of the thioketone bonding to gold atoms. This arrangement results in the formation of a rigid five-membered ring (Au–Cu–N–C–S) and a more compact Cu–Au core. The terminal benzyl groups exhibit distinctive spatial orientations, reflecting their considerable rotational freedom, as the nanoclusters exist as discrete molecules. Notably, when the clusters aggregate into a film state, the outer benzyl groups of distinct clusters exhibit a propensity to interact, leading to the creation of intercluster C–H···π and π···π interactions.^37^ As shown in Figure S2, these noncovalent interactions play a crucial role in guiding the nanocluster self-assembly, resulting in a monoclinic crystal system. Besides, these interactions coupled with electron delocalization traits inherent in the benzyl groups hold considerable promise in enhancing the conductivity of Cu_2_Au_2_(R/S-BTT)_4_ films. Such enhanced conductivity holds paramount importance in ensuring the successful fabrication of high-performance LEDs based on pure clusters. Additionally, we investigated the molecular structure stability of Cu_2_Au_2_(S-BTT)_4_ in solution through nuclear magnetic resonance (NMR). As illustrated in Figures S3 and S4, no resonance signal corresponding to free S-BTT ligands was detected in the NMR spectra of Cu_2_Au_2_(S-BTT)_4_. The absence of such signals implies the robust stability of Cu_2_Au_2_(S-BTT)_4_.

In pursuit of high-performance LEDs, the other critical factor is to use an emitting material with a high ϕ_PL_ in the film state. Both Cu_2_Au_2_(R-BTT)_4_ and Cu_2_Au_2_(S-BTT)_4_ display desirable AIEE behavior, as evidenced by the distinct enhancement of their photoluminescence when their solutions were transferred to films (Figure 1b). As shown in Figure 1c, the ϕ_PL_ of Cu_2_Au_2_(R-BTT)_4_ and Cu_2_Au_2_(S-BTT)_4_ increases significantly from 7 and 13 to 94 and 89%, respectively. (The slight decrease in ϕ_PL_ for Cu_2_Au_2_(S-BTT)_4_ compared to Cu_2_Au_2_(R-BTT)_4_ can be attributed to variations in the purity of raw materials.) This remarkable AIEE behavior originates from the pronounced restriction of molecular motions. More precisely, upon self-assembly into a film state, the rotational and vibrational movements of the terminal groups in Cu_2_Au_2_(R/S-BTT)_4_ experience significant constraints due to spatial confinement. This restriction, caused by the intercluster C–H···π and π···π interactions, is promising to reduce the nonradiative recombination of excitons. Furthermore, upon the transformation of solutions into films, the ultraviolet–visible (UV–vis) absorption spectra (Figure S5) exhibit a significant blue shift, whereas the PL spectra (Figure 1d) show a slight red shift, resulting in an expanded Stokes shift. The large Stokes shift of emitting materials is a desirable characteristic when employed in LEDs, as it can diminish undesired photon reabsorption.^38^ Meanwhile, subtle variations are observed in the emission peaks and absorption spectra of Cu_2_Au_2_(R-BTT)_4_ and Cu_2_Au_2_(S-BTT)_4_, which may arise from slight differences in the electronic structures that are influenced by their configuration at the chiral center. The three-dimensional excitation–emission matrix (3D-EEM) luminance spectra (Figure S6) show that the PL emission peaks are independent of excitation and only slightly shift as the solutions are transferred to films, indicating that the clusters possess a similar exciton recombination process in different states. In contrast, the PL excitation (PLE) spectra (Figure 1d) exhibit a remarkable blue shift, implying that the excited states change due to aggregation. To explore their impact on the PL properties, the PL stability and lifetime were investigated. Figure 1e demonstrates the PL intensity evolutions of the clusters under constant excitation, where the *T*_90_ (time of the PL intensity decreasing to 90% of initial value) of Cu_2_Au_2_(R-BTT)_4_ and Cu_2_Au_2_(S-BTT)_4_ increases significantly from 1.83 and 0.67 to 87.00 and 25.83 min, respectively, as their solutions are transferred to films. Additionally, the time-resolved photoluminescence (TRPL) decay curves exhibit a remarkable augmentation of the PL lifetime by several orders of magnitude (Figure 1f) upon the transition of their solutions into film states. This pronounced extension is poised to considerably amplify the radiative recombination ratio, thereby underscoring a significant potential for the development of high-efficiency LED devices.

### Chirality and Circularly Polarized Photoluminescence (CPPL) Property

The ultraviolet–visible circular dichroism (UV-CD) spectra of Cu_2_Au_2_(R/S-BTT)_4_ show a mirror-image relationship with opposite Cotton effects (Figure 1g), which is distinctly different from the chiral ligands of R/S-BTT (Figure S7), indicating the chiral nature of the synthesized clusters. Specifically, Cu_2_Au_2_(R-BTT)_4_ exhibits positive Cotton effects at 265 and 351 nm and a negative Cotton effect at 299 nm, suggesting a dextrorotatory structure. In contrast, Cu_2_Au_2_(S-BTT)_4_ shows opposite Cotton effects, indicating a levorotatory structure. The UV-CD spectra are consistent with single-crystal X-ray crystallography. To evaluate the CPPL properties of the clusters, we investigated solution-processed cluster films using CPL spectroscopy. Figure 1h illustrates the symmetrical CPPL signals of Cu_2_Au_2_(R/S-BTT)_4_ films in the range of 525–700 nm. To quantitatively access the CPL property, the dissymmetry factor (*g*, *g* = 2 × (*I*_R_ – *I*_L_)/ (*I*_R_ + *I*_L_), where *I*_R_ and *I*_L_ represent the intensity of right- and left-CPL, respectively) was introduced. As depicted in Figure 1i, the *g*_PL_ values of Cu_2_Au_2_(R-BTT)_4_ and Cu_2_Au_2_(S-BTT)_4_ films are approximately 1.0 × 10^–3^ and −1.5 × 10^–3^, respectively, which are in the same order of magnitude as those of chiral organic molecules.

### CP-LEDs Based on Pure MNCs

**Figure 2:**
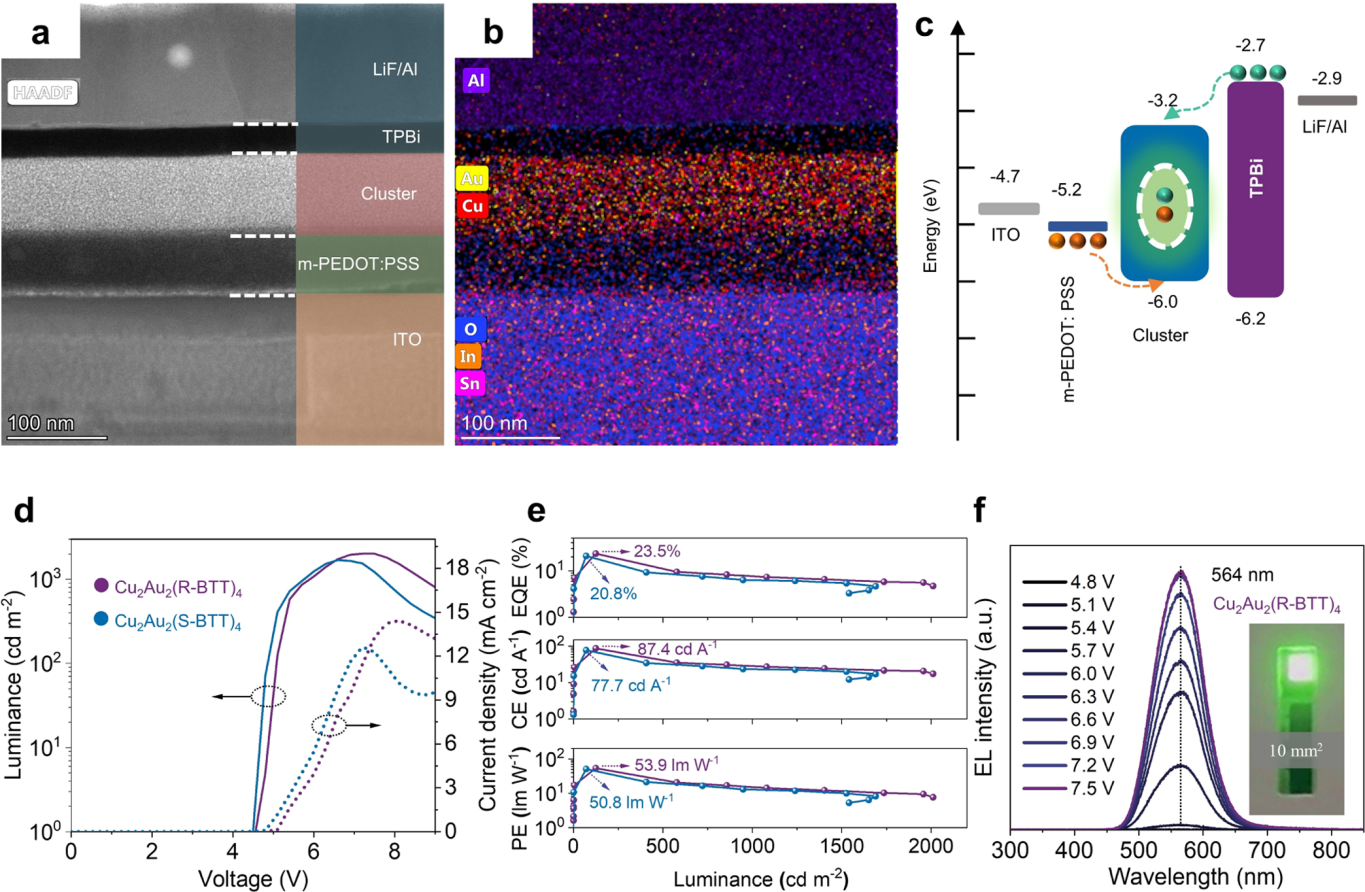
Device structure and performances of CP-LEDs based on pure Cu_2_Au_2_(R/S-BTT)_4_. (a) TEM image and (b) corresponding EDS of the cross-sectional CP-LED device based on Cu_2_Au_2_(R-BTT)_4_. (c) Energy band alignment of the CP-LED device. (d) Luminance–voltage–current density curves and (e) EQE, CE, and PE versus luminance curves of the CP-LED devices based on Cu_2_Au_2_(R/S-BTT)_4_. (f) EL spectra of the CP-LED devices based on Cu_2_Au_2_(R-BTT)_4_ with increased driving voltage and photograph of the operational device (inset).

CP-LED devices were fabricated in a standard “sandwich” structure, consisting of indium tin oxide (ITO)/modified poly(3,4-ethylenedioxythiophene)-poly(styrenesulfonate) (m-PEDOT:PSS)/Cu_2_Au_2_(R/S-BTT)_4_/2,2′,2″-(1,3,5-benzenetriyl)-tris(1-phenyl-1-*H*-benzimidazole) (TPBi)/lithium fluoride (LiF)/aluminum (Al). The image of cross-sectional CP-LED device and corresponding energy dispersive spectroscopy (EDS) captured by transmission electron microscopy (TEM) are presented in Figure 2a,2b, respectively. Both images confirm that the device was fabricated according to the designed structure. Furthermore, the scanning electron microscopy (SEM) image and corresponding EDS display a dense and uniform cluster film that was achieved through a solution processing method (Figure S8a,b). The atomic force microscopy (AFM) image confirms that the prepared film is smooth with low roughness (Figure S8c). Figure 2c demonstrates the energy alignment of the CP-LED based on Cu_2_Au_2_(R-BTT)_4_. The highest occupied molecular orbital (HOMO) of −6.0 eV is obtained from analysis of the ultraviolet photoelectron spectroscopy (UPS) results (Figure S9). The band gap is calculated from the optical binding energy obtained from the UV–vis absorption in Figure S5. To gain deeper insights into the excited states, we investigated the distribution of electronic charge densities within the frontier molecular orbitals, utilizing density functional theory (DFT) calculations. As illustrated in Figure S10, the energy levels of the HOMO and the lowest unoccupied molecular orbital (LUMO) are consistent with the experimental observations. Notably, the electronic charge densities associated with the HOMO and LUMO of Cu_2_Au_2_(R/S-BTT)_4_ are predominantly localized in the regions encompassing the S/N atoms and central metal atoms, which significantly suggests that the emission could arise from charge transfer excited states. To visually analyze the transition characteristics of the excited states, we further investigated the natural transition orbitals (NTOs) of Cu_2_Au_2_(R/S-BTT)_4_. As shown in Figure S11, the outcomes reveal nearly identical contours of the highest occupied (“hole”) and the lowest unoccupied (“particle”) NTOs for first singlet (S_0_ → S_1_) and first triplet (S_0_ → T_1_) transitions, which are mainly localized on metal cores and ligand motifs, respectively. These transitions correspond to HOMO → LUMO, indicating that both S_1_ and T_1_ are dominated by HOMO → LUMO transitions. Moreover, the metal core-centralized “holes” and ligand-centralized “particles” ensure that the S_1_ and T_1_ excited states predominantly arise from metal-to-ligand charge transfer (MLCT), leading to negligible nonradiative triplet cluster-centered components.

We first examined the effect of the emitter thickness on device performance, as it has a significant impact on radiative recombination centers and light outcoupling efficiency.^39^ The thickness of the Cu_2_Au_2_(R-BTT)_4_ films, prepared using different precursor concentrations, is detailed in Figure S12a–e. As summarized in Figure S12f, the film thickness exhibited a nearly linear relationship with precursor concentration within the range of 9–21 mg mL^–1^. Consequently, the emitting layer’s thickness can be precisely controlled by adjusting the precursor concentrations. Figure S13 presents the performance of CP-LEDs manufactured with varying concentrations of Cu_2_Au_2_(R-BTT)_4_. As summarized in Table S2, the device fabricated with 15 mg mL^–1^ Cu_2_Au_2_(R-BTT)_4_ achieved an optimal thickness of approximately 60 nm, resulting in superior luminance and EQE. Utilizing this optimal precursor concentration, we proceeded to fabricate devices based on pure Cu_2_Au_2_(R/S-BTT)_4_. The luminance–voltage–current density (*L*–*V*–*J*) curves (Figure 2d) show that the CP-LEDs based on Cu_2_Au_2_(R-BTT)_4_ and Cu_2_Au_2_(S-BTT)_4_ reach a peak luminance of 2010 and 1670 cd m^–2^, respectively, at a driving voltage of 7.2 and 6.6 V. Figure 2e depicts the efficiency curves versus luminance of the CP-LEDs, in which those based on Cu_2_Au_2_(R-BTT)_4_ and Cu_2_Au_2_(S-BTT)_4_ achieve a maximum EQE (EQE_max_) of 23.5 and 20.8%, respectively, corresponding to a maximum current efficiency (CE_max_) of 87.4 and 77.7 cd A^–1^ and maximum power efficiency (PE_max_) of 53.9 and 50.8 lm W^–1^. To ensure the accuracy of the efficiencies, calibration was preformed using a commercially available inorganic LED. By comparing our test results with those provided by the device manufacturers, it is evident that the observed discrepancy is less than 1% (Figure S14). The static EQE_max_ values of 60 devices are recorded in Figure S15a, which indicates excellent reproducibility of high-efficiency CP-LEDs. The electroluminescence (EL) spectra of the CP-LEDs based on Cu_2_Au_2_(R-BTT)_4_ and Cu_2_Au_2_(S-BTT)_4_ are shown in Figures 2f and S15b, respectively. Both devices emit stably at 564 and 562 nm, respectively, under different driving voltages (the inset in Figure 2f shows an operational device with an active area of 9.98 mm^2^). Additionally, the emission coordinates of the CP-LEDs based on Cu_2_Au_2_(R-BTT)_4_ and Cu_2_Au_2_(S-BTT)_4_ are determined to be (0.395, 0.572) and (0.394, 0.574) on the Commission Internationale de l’Eclairage (CIE) chromaticity diagram (Figure S15c), indicating a high degree of color purity of the emissions. Additionally, the operational stability of the CP-LEDs was evaluated under a constant current density of 0.2 mA cm^–2^, and the evolutions of their voltage, luminance, and EQE are recorded in Figure S16. The CP-LEDs based on Cu_2_Au_2_(R-BTT)_4_ and Cu_2_Au_2_(S-BTT)_4_ achieved half lifetimes of 395 and 251 s, respectively.

### CP-LEDs Based on MNCs with Host Incorporation

**Figure 3:**
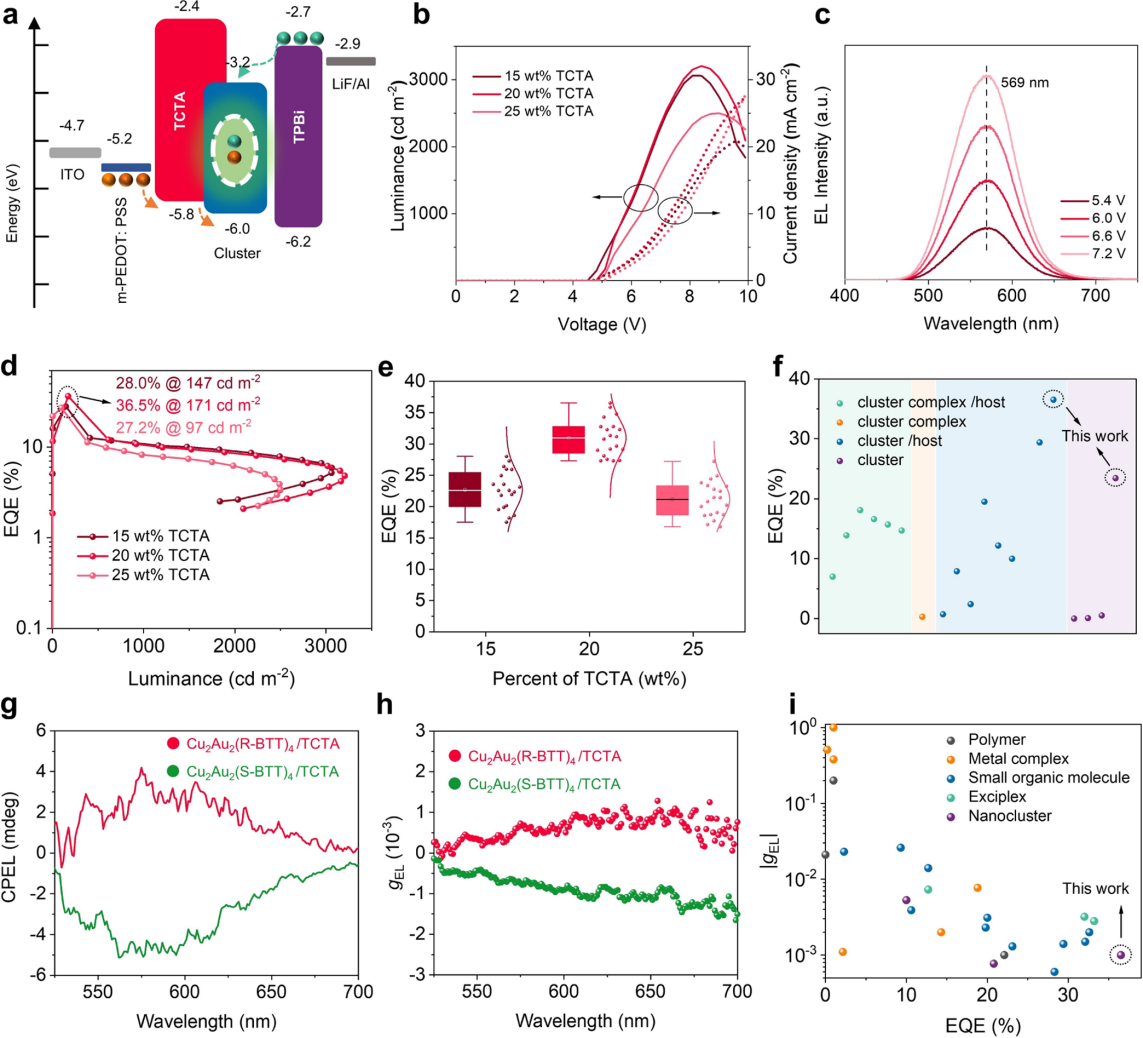
Performances of CP-LEDs based on a cluster with TCTA incorporating. (a) Energy band alignment of the CP-LED with TCTA incorporation. (b) Luminance–voltage–current density curves of the CP-LEDs with different weight ratios of TCTA. (c) EL spectra of the CP-LED with 20 wt % TCTA incorporation. (d) EQE–luminance curves and (e) statical maximum EQEs of the CP-LEDs with different weight ratios of TCTA. (f) Summary of the EQEs of the LEDs using clusters or cluster complexes as parts of the emitting center (data in the figure are listed in Table S4). (g) CPEL spectra and (h) *g*_EL_ versus wavelength curves of the CP-LEDs based on Cu_2_Au_2_(R/S-BTT)_4_ with 20 wt % TCTA incorporation. (i) Summary of EQEs and |*g*_EL_| for CP-LEDs in the literature and our work (data in the figure are listed in Table S5).

In our pursuit of enhancing CP-LED performance, we introduced the commonly employed host material TCTA into the clusters. As shown in Figure S17, the absorption and PL spectra of Cu_2_Au_2_(R-BTT)_4_ films, with TCTA incorporation not exceeding 25 wt %, exhibited negligible alterations, indicating that the emissions still originate from the clusters. Notably, the energy alignment illustrated in Figure 3a suggests that TCTA serves as both a hole injection and electron-blocking material, holding promise for exciton confinement and reduction of nonradiative recombination. The *L*–*V*–*J* curves of the devices with different ratios of TCTA incorporated (Figure 3b) exhibit a distinguished increase of the luminance and current density compared to those without TCTA (Figure 2d). The maximum luminance of the devices with 15, 20, and 25 wt % TCTA incorporation reaches 3063, 3206, and 2499 cd m^–2^, respectively. The EL spectra in Figure 3c reveal a slight emission red shift, shifting from 564 to 569 nm upon the incorporation of TCTA into Cu_2_Au_2_(R-BTT)_4_.

Figure 3d offers insights into the EQE–luminance relationship, where peak EQEs of 28, 36.5, and 27.2% are achieved at the luminance of 147, 171, and 97 cd m^–2^, respectively. The corresponding statistical maximum EQEs are presented in Figure 3e, highlighting that CP-LEDs incorporating 20 wt % TCTA demonstrate exceptional EQE performance, positioning them as champion devices. The CP-LEDs based on Cu_2_Au_2_(S-BTT)_4_ with 20 wt % TCTA incorporation were also fabricated and evaluated, which exhibit analogous improvements to those based on Cu_2_Au_2_(R-BTT)_4_. As shown in Figure S18a,S18b, the luminance and EQE were notably improved to 3197 cd m^–2^ and 32.7%, respectively. The EL spectra in Figure S18c exhibit a stable emission peak at 566 nm under different driving voltages. The performances of the most optimal devices are summarized in Table S3. To evaluate the performance of our cluster-based LEDs, we compiled a summary of LEDs employing clusters or cluster complexes as integral components of the emitting center, as presented in Table S4. Their respective EQEs are depicted in Figure 3f. We noted that our devices have reached record EQEs of 23.5 and 36.5% in the LEDs based on the pure cluster and cluster with host incorporation, respectively. These exceptional EQEs make our cluster-based LEDs competitive with other emerging LED technologies, such as organic LEDs, quantum dot LEDs, and perovskite LEDs (Figure S19).

The CPEL of the best-performing devices was additionally investigated by CPL spectroscopy. Figure 3g depicts the symmetrical CPEL signals spanning the range from 525 to 700 nm for the CP-LEDs based on Cu_2_Au_2_(R/S-BTT)_4_ with 20 wt % TCTA incorporation. As shown in Figure 3h, their |*g*_EL_| values are approximate 1.0 × 10^–3^, aligning with the levels observed in most reported CP-LEDs based on the polymer, small organic molecule, and exciplex. Furthermore, to obtain the separated left-handed and right-handed circularly polarized light spectra, we constructed homemade equipment. As illustrated in Figure S20a, the EL beam including both right- and left-CPEL is passed through a quarter-wave plate, which changes it into mutually perpendicular linearly polarized lights. These light beams can then be filtered by using a linear polarizer. The right- and left-CPEL spectra are shown in Figure S20b,c, respectively. Table S5 and Figure 3i provide a summary of the EQEs and |*g*_EL_| values of recently reported CP-LEDs. Our devices achieved a record EQE in the realm of CP-LEDs, while attaining |*g*_EL_| values comparable to those of other CP-LEDs, except for a few that rely on polymers and metal complexes and exhibit very low EQEs.

### Transient Absorption (TA) Spectra, TRPL Decay Curves, and Phosphorescence Emission Process

**Figure 4:**
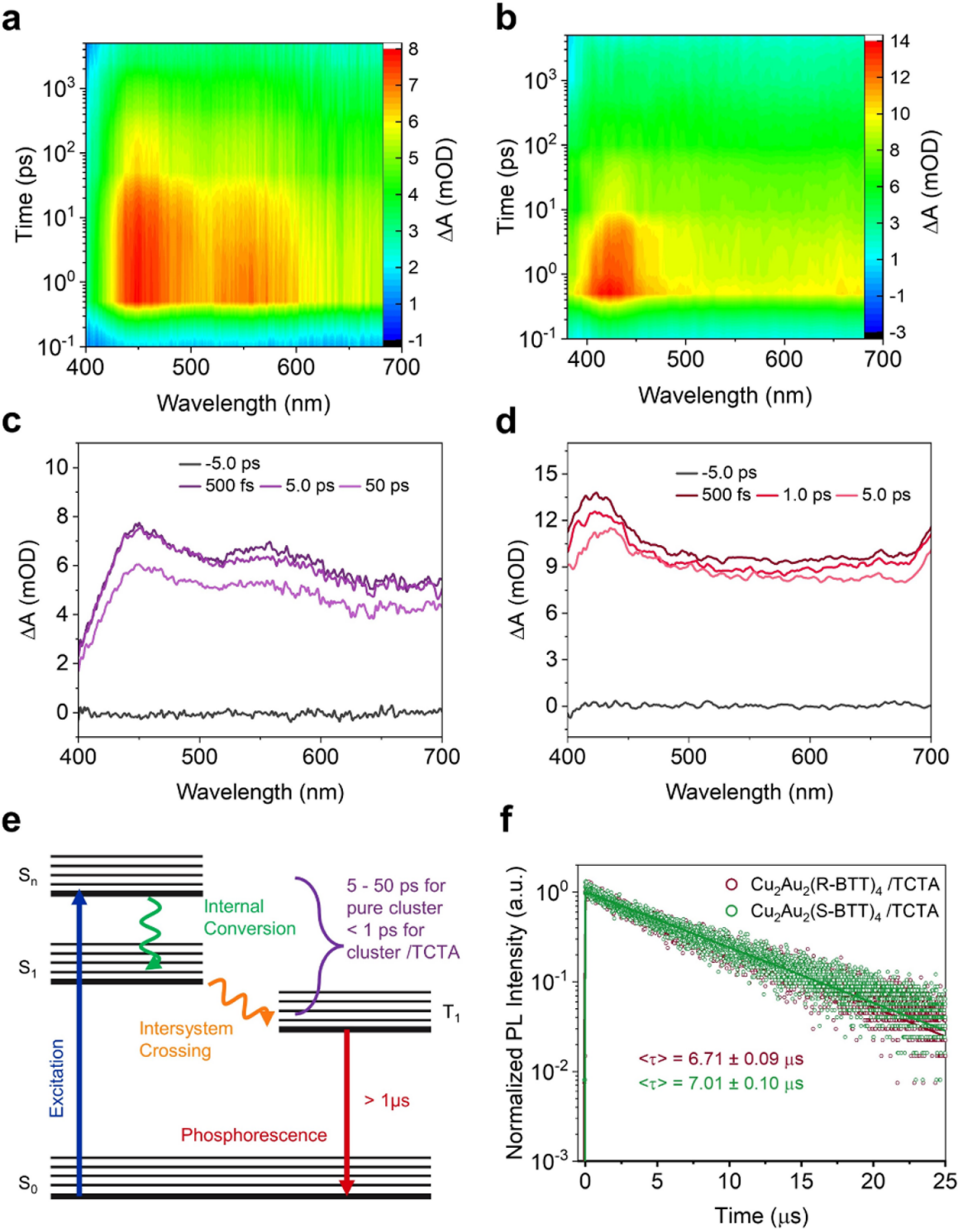
Transient absorption (TA) spectra, TRPL decay curves, and phosphorescence emission process. 2D pseudocolor TA spectra of (a) pure Cu_2_Au_2_(R-BTT)_4_ and (b) Cu_2_Au_2_(R-BTT)_4_ with 20 wt % TCTA. TA spectra at different delay times of (c) pure Cu_2_Au_2_(R-BTT)_4_ and (d) Cu_2_Au_2_(R-BTT)_4_ with 20 wt % TCTA. (e) Schematic of the phosphorescence emission process. (f) TRPL decay curves of Cu_2_Au_2_(R/S-BTT)_4_ with 20 wt % TCTA incorporation.

To examine the differences in the ultrafast electron dynamics of the cluster films with and without TCTA incorporation, we performed femtosecond transient absorption (fs-TA) spectroscopy. Under 365 nm pumped laser excitation, as the two-dimensional (2D) pseudocolor fs-TA spectra shown in Figure 4a, the Cu_2_Au_2_(R-BTT)_4_ film exhibits two photoinduced absorption (PA) signals, centered at 450 and 550 nm, attributed to the excited-state absorption (ESA). Upon TCTA incorporation, the PA signal undergoes narrowing and its peak shifts to 425 nm (Figure 4b), indicating heightened energy level organization arising from the molecular interactions between TCTA and Cu_2_Au_2_(R-BTT)_4_. To delve into the decay kinetics of the PA signals, we extracted TA spectra at various delay times. Notably, the Cu_2_Au_2_(R-BTT)_4_ film exhibits negligible decay over a 5 ps time scale (Figure 4c), whereas a rapid decay transpires within just 1 ps for the film with TCTA incorporation (Figure 4d). The identical phenomenon is observed in the TA spectra of Cu_2_Au_2_(S-BTT)_4_ films with or without TCTA incorporation, as demonstrated in Figure S21. As schematic illustrated in Figure 4e, the kinetic traces of the PA signals are assigned to the internal conversion (IC) from S*_n_* to S_1_ state coupled with the intersystem crossing (ISC) from S_1_ to T_1_, as no additional relaxation is observed. The shorter decay time indicates faster IC and ISC processes for cluster films with TCTA incorporation. After the rapid IC and ISC processes, the excitons recombine from a long-lived triplet excited state to the ground state, emitting phosphorescence. Additionally, TCTA incorporation leads to prolonged PL lifetimes, increasing from 3.89 and 3.92 μs (as seen in Figure 1f) to 6.71 and 7.01 μs for the Cu_2_Au_2_(R-BTT)_4_ and Cu_2_Au_2_(R-BTT)_4_ films, respectively (Figure 4f). The rapid IC and ISC processes, coupled with long PL lifetimes, are promising to increase the ratio of radiative recombination and bolster high-efficiency LED fabrication.

## Conclusions

In summary, we advanced high-efficiency CP-LEDs through novel enantiopure MNC films with high electronic quality and AIEE. The EQEs of the CP-LEDs based on the MNCs were dramatically improved (EQE = 23.5%, compared to < 10% in previous reports). Adding just 20 wt % TCTA as a host further optimized band alignment, enabling rapid singlet–triplet transfer and a record EQE of 36.5% for both CP-LEDs and MNC-LEDs, with |*g*_EL_| ∼ 1.0 × 10^–3^. These findings underscore the remarkable potential of chiral MNCs to propel the development of high-efficiency CP-LEDs.

## Supporting Information Available

Precursors and film preparation; CP-LED device fabrication; single-crystal XRD measurements; photoluminescence and absorption characterizations; femtosecond transient absorption measurements; time-resolved photoluminescence decay measurements; transmission electron microscopy measurements; and performance evaluation of the LED (PDF)

## References

[ref1] Sherson J. F.; Krauter H.; Olsson R. K.; Julsgaard B.; Hammerer K.; Cirac I.; Polzik E. S. Quantum teleportation between light and matter. Nature 2006, 443 (7111), 557–560. 10.1038/nature05136. 17024089

[ref2] Yang Y.; da Costa R. C.; Fuchter M. J.; Campbell A. J. Circularly polarized light detection by a chiral organic semiconductor transistor. Nat. Photonics 2013, 7 (8), 634–638. 10.1038/nphoton.2013.176.

[ref3] Zhang X. G.; Yu Q.; Jiang W. X.; Sun Y. L.; Bai L.; Wang Q.; Qiu C. W.; Cui T. J. Polarization-Controlled Dual-Programmable Metasurfaces. Adv. Sci. 2020, 7 (11), 1903382 10.1002/advs.201903382. PMC7284210 32537403

[ref4] Lee G. Y.; Hong J. Y.; Hwang S.; Moon S.; Kang H.; Jeon S.; Kim H.; Jeong J. H.; Lee B. Metasurface eyepiece for augmented reality. Nat. Commun. 2018, 9 (1), 4562 10.1038/s41467-018-07011-5. 30385830 PMC6212528

[ref5] Stachelek P.; MacKenzie L.; Parker D.; Pal R. Circularly polarised luminescence laser scanning confocal microscopy to study live cell chiral molecular interactions. Nat. Commun. 2022, 13 (1), 553 10.1038/s41467-022-28220-z. 35087047 PMC8795401

[ref6] Wang Q.; Plum E.; Yang Q.; Zhang X.; Xu Q.; Xu Y.; Han J.; Zhang W. Reflective chiral meta-holography: multiplexing holograms for circularly polarized waves. Light: Sci. Appl. 2018, 7, 25 10.1038/s41377-018-0019-8. 30839596 PMC6106984

[ref7] Crassous J.; Fuchter M. J.; Freedman D. E.; Kotov N. A.; Moon J.; Beard M. C.; Feldmann S. Materials for chiral light control. Nat. Rev. Mater. 2023, 8, 365–371. 10.1038/s41578-023-00543-3.

[ref8] Zhang D. W.; Li M.; Chen C. F. Recent advances in circularly polarized electroluminescence based on organic light-emitting diodes. Chem. Soc. Rev. 2020, 49 (5), 1331–1343. 10.1039/C9CS00680J. 31999286

[ref9] Wan L.; Liu Y.; Fuchter M. J.; Yan B. Anomalous circularly polarized light emission in organic light-emitting diodes caused by orbital–momentum locking. Nat. Photonics 2023, 17 (2), 193–199. 10.1038/s41566-022-01113-9.

[ref10] Wang X.; Ma S.; Zhao B.; Deng J. Frontiers in Circularly Polarized Phosphorescent Materials. Adv. Funct. Mater. 2023, 33, 2214364 10.1002/adfm.202214364.

[ref11] Deng Y.; Wang M.; Zhuang Y.; Liu S.; Huang W.; Zhao Q. Circularly polarized luminescence from organic micro-/nano-structures. Light: Sci. Appl. 2021, 10 (1), 76 10.1038/s41377-021-00516-7. 33840811 PMC8039044

[ref12] Zinna F.; Pasini M.; Galeotti F.; Botta C.; Di Bari L.; Giovanella U. Design of Lanthanide-Based OLEDs with Remarkable Circularly Polarized Electroluminescence. Adv. Funct. Mater. 2017, 27, 1603719 10.1002/adfm.201603719.

[ref13] Lunkley J. L.; Shirotani D.; Yamanari K.; Kaizaki S.; Muller G. Extraordinary circularly polarized luminescence activity exhibited by cesium tetrakis(3-heptafluoro-butylryl-(+)-camphorato) Eu(III) complexes in EtOH and CHCl3 solutions. J. Am. Chem. Soc. 2008, 130 (42), 13814–13815. 10.1021/ja805681w. 18816117 PMC2701347

[ref14] Zinna F.; Arrico L.; Funaioli T.; Di Bari L.; Pasini M.; Botta C.; Giovanella U. Modular chiral Eu(iii) complexes for efficient circularly polarized OLEDs. J. Mater. Chem. C 2022, 10 (2), 463–468. 10.1039/D1TC05023K.

[ref15] Xu Y.; Wang Q.; Cai X.; Li C.; Wang Y. Highly Efficient Electroluminescence from Narrowband Green Circularly Polarized Multiple Resonance Thermally Activated Delayed Fluorescence Enantiomers. Adv. Mater. 2021, 33 (21), e2100652 10.1002/adma.202100652. 33864284

[ref16] Liao X. J.; Pu D.; Yuan L.; Tong J.; Xing S.; Tu Z. L.; Zuo J. L.; Zheng W. H.; Zheng Y. X. Planar Chiral Multiple Resonance Thermally Activated Delayed Fluorescence Materials for Efficient Circularly Polarized Electroluminescence. Angew. Chem., Int. Ed. 2023, 62 (6), e202217045 10.1002/anie.202217045. 36517419

[ref17] Yang S. Y.; Wang Y. K.; Peng C. C.; Wu Z. G.; Yuan S.; Yu Y. J.; Li H.; Wang T. T.; Li H. C.; Zheng Y. X.; et al. Circularly Polarized Thermally Activated Delayed Fluorescence Emitters in Through-Space Charge Transfer on Asymmetric Spiro Skeletons. J. Am. Chem. Soc. 2020, 142 (41), 17756–17765. 10.1021/jacs.0c08980. 33021373

[ref18] Yang Y.; da Costa R. C.; Smilgies D. M.; Campbell A. J.; Fuchter M. J. Induction of circularly polarized electroluminescence from an achiral light-emitting polymer via a chiral small-molecule dopant. Adv. Mater. 2013, 25 (18), 2624–2628. 10.1002/adma.201204961. 23554220 PMC3659407

[ref19] Wang Y. F.; Li M.; Teng J. M.; Zhou H. Y.; Zhao W. L.; Chen C. F. Chiral TADF-Active Polymers for High-Efficiency Circularly Polarized Organic Light-Emitting Diodes. Angew. Chem., Int. Ed. 2021, 60 (44), 23619–23624. 10.1002/anie.202110794. 34490710

[ref20] Geng Z.; Zhang Y.; Zhang Y.; Li Y.; Quan Y.; Cheng Y. Circularly polarized electroluminescence from an achiral fluorophore induced by co-assembly with chiral polymers. J. Mater. Chem. C 2021, 9 (36), 12141–12147. 10.1039/D1TC01948A.

[ref21] Chen Z.; Huang M.; Zhong C.; Cao X.; Xie G.; Gong S.; Yang C. Cascade Chirality Transfer Through Diastereomeric Interaction Enables Efficient Circularly Polarized Electroluminescence. Adv. Funct. Mater. 2023, 33 (21), 2215179 10.1002/adfm.202215179.

[ref22] Lu G.; Wu Z. G.; Wu R.; Cao X.; Zhou L.; Zheng Y. X.; Yang C. Semitransparent Circularly Polarized Phosphorescent Organic Light-Emitting Diodes with External Quantum Efficiency over 30% and Dissymmetry Factor Close to 10–2. Adv. Funct. Mater. 2021, 31 (36), 2102898 10.1002/adfm.202102898.

[ref23] Song J.; Xiao H.; Fang L.; Qu L.; Zhou X.; Xu Z. X.; Yang C.; Xiang H. Highly Phosphorescent Planar Chirality by Bridging Two Square-Planar Platinum(II) Complexes: Chirality Induction and Circularly Polarized Luminescence. J. Am. Chem. Soc. 2022, 144 (5), 2233–2244. 10.1021/jacs.1c11699. 35048693

[ref24] Jin R.; Zeng C.; Zhou M.; Chen Y. Atomically Precise Colloidal Metal Nanoclusters and Nanoparticles: Fundamentals and Opportunities. Chem. Rev. 2016, 116 (18), 10346–10413. 10.1021/acs.chemrev.5b00703. 27585252

[ref25] Liu L.; Bai B.; Yang X.; Du Z.; Jia G. Anisotropic Heavy-Metal-Free Semiconductor Nanocrystals: Synthesis, Properties, and Applications. Chem. Rev. 2023, 123 (7), 3625–3692. 10.1021/acs.chemrev.2c00688. 36946890

[ref26] Matus M. F.; Häkkinen H. Understanding ligand-protected noble metal nanoclusters at work. Nat. Rev. Mater. 2023, 8 (6), 372–389. 10.1038/s41578-023-00537-1.

[ref27] Song Y.; Li Y.; Zhou M.; Liu X.; Li H.; Wang H.; Shen Y.; Zhu M.; Jin R. Ultrabright Au@Cu14 nanoclusters: 71.3% phosphorescence quantum yield in non-degassed solution at room temperature. Sci. Adv. 2021, 7 (2), eabd2091 10.1126/sciadv.abd2091. 33523969 PMC7787487

[ref28] Yao Q.; Liu L.; Malola S.; Ge M.; Xu H.; Wu Z.; Chen T.; Cao Y.; Matus M. F.; Pihlajamaki A.; et al. Supercrystal engineering of atomically precise gold nanoparticles promoted by surface dynamics. Nat. Chem. 2023, 15 (2), 230–239. 10.1038/s41557-022-01079-9. 36357788

[ref29] Ni J.; Zhong C.; Li L.; Su M.; Wang X.; Sun J.; Chen S.; Duan C.; Han C.; Xu H. Deep-Blue Electroluminescence from Phosphine-Stabilized Au(3) Triangles and Au(3) Ag Pyramids. Angew. Chem. 2022, 134 (47), e202213826 10.1002/anie.202213826. 36202754

[ref30] Wang J. J.; Chen C.; Chen W. G.; Yao J. S.; Yang J. N.; Wang K. H.; Yin Y. C.; Yao M. M.; Feng L. Z.; Ma C.; et al. Highly Luminescent Copper Iodide Cluster Based Inks with Photoluminescence Quantum Efficiency Exceeding 98. J. Am. Chem. Soc. 2020, 142 (8), 3686–3690. 10.1021/jacs.9b12908. 32045234

[ref31] Yu H.; Rao B.; Jiang W.; Yang S.; Zhu M. The photoluminescent metal nanoclusters with atomic precision. Coord. Chem. Rev. 2019, 378, 595–617. 10.1016/j.ccr.2017.12.005.

[ref32] Zhang N.; Li Y.; Han S.; Wei Y.; Hu H.; Huo R.; Duan C.; Zhang J.; Han C.; Xie G.; et al. Cluster Light-Emitting Diodes Containing Copper Iodine Cube with 100% Exciton Utilization Using Host-Cluster Synergy. Angew. Chem., Int. Ed. 2023, 62 (27), e202305018 10.1002/anie.202305018. 37129949

[ref33] Xie M. C.; Han C. M.; Liang Q. Q.; Zhang J.; Xie G. H.; Xu H. Highly efficient sky blue electroluminescence from ligand-activated copper iodide clusters: Overcoming the limitations of cluster light-emitting diodes. Sci. Adv. 2019, 5 (6), eaav9857 10.1126/sciadv.aav9857. 31245537 PMC6588361

[ref34] Niesen B.; Rand B. P. Thin film metal nanocluster light-emitting devices. Adv. Mater. 2014, 26 (9), 1446–1449. 10.1002/adma.201304725. 24734300

[ref35] Koh T. W.; Hiszpanski A. M.; Sezen M.; Naim A.; Galfsky T.; Trivedi A.; Loo Y. L.; Menon V.; Rand B. P. Metal nanocluster light-emitting devices with suppressed parasitic emission and improved efficiency: exploring the impact of photophysical properties. Nanoscale 2015, 7 (20), 9140–9146. 10.1039/C5NR01332A. 25926355

[ref36] Wang J. J.; Zhou H. T.; Yang J. N.; Feng L. Z.; Yao J. S.; Song K. H.; Zhou M. M.; Jin S.; Zhang G.; Yao H. B. Chiral Phosphine-Copper Iodide Hybrid Cluster Assemblies for Circularly Polarized Luminescence. J. Am. Chem. Soc. 2021, 143 (29), 10860–10864. 10.1021/jacs.1c05476. 34279083

[ref37] Huang R. W.; Song X.; Chen S.; Yin J.; Maity P.; Wang J.; Shao B.; Zhu H.; Dong C.; Yuan P.; et al. Radioluminescent Cu-Au Metal Nanoclusters: Synthesis and Self-Assembly for Efficient X-ray Scintillation and Imaging. J. Am. Chem. Soc. 2023, 145 (25), 13816–13827. 10.1021/jacs.3c02612. 37335564

[ref38] Perego J.; Bezuidenhout C. X.; Villa I.; Cova F.; Crapanzano R.; Frank I.; Pagano F.; Kratochwill N.; Auffray E.; Bracco S.; et al. Highly luminescent scintillating hetero-ligand MOF nanocrystals with engineered Stokes shift for photonic applications. Nat. Commun. 2022, 13 (1), 3504 10.1038/s41467-022-31163-0. 35715391 PMC9205964

[ref39] Zhao L.; Lee K. M.; Roh K.; Khan S. U. Z.; Rand B. P. Improved Outcoupling Efficiency and Stability of Perovskite Light-Emitting Diodes using Thin Emitting Layers. Adv. Mater. 2019, 31 (2), e1805836 10.1002/adma.201805836. 30412319

