# Supplementary material for: High-Efficiency Circularly Polarized Light-Emitting Diodes Based on Chiral Metal Nanoclusters
Source: J Am Chem Soc. 2024 Feb 5;146(6):4144–52. doi: 10.1021/jacs.3c13065 (PMC10870708; doi:10.1021/jacs.3c13065)
Supplement: Supplementary file 1 — ja3c13065_si_001.pdf [file ja3c13065_si_001.pdf]

Supporting Information

High-efficiency circularly polarized light-emitting diodes based on chiral metal nanoclusters

Jianxun Lu,[1, †] Bingyao Shao,[1, †] Ren-Wu Huang,[2, †] Luis Gutiérrez-Arzaluz,[1,3] Shulin Chen,[1] Zhen Han,[2] Jun Yin,[4] Hongwei Zhu,[1] Sergey Dayneko,[5] Mohamed Nejib Hedhili,[6] Xin Song,[1] Peng Yuan,[1] Chunwei Dong,[1] Renqian Zhou,[1] Makhsud I. Saidaminov,[5] Shuang-Quan Zang,[2] Omar F. Mohammed,[1, 3, *] and Osman M. Bakr[1, *]

[1] *Division of Physical Science and Engineering, KAUST Catalysis Center (KCC), King Abdullah University of Science and Technology, Thuwal 23955-6900, Kingdom of Saudi Arabia*

[2] *Key Laboratory of Crystalline Molecular Functional Materials, Henan International Joint Laboratory of Tumor Theranostical Cluster Materials, Green Catalysis Center, and College of Chemistry, Zhengzhou University, Zhengzhou 450001, China.*

[3] *Division of Physical Science and Engineering, Advanced Membranes and Porous Materials Center (AMPM), King Abdullah University of Science and Technology, Thuwal 23955-6900, Kingdom of Saudi Arabia*

[4] *Department of Applied Physics, The Hong Kong Polytechnic University, 999077 Hong Kong, P.R. China*

[5] *Department of Electrical and Computer Engineering, University of Victoria, 3800 Finnerty Rd, Victoria, BC, V8P 5C2 Canada*

[6] *The Imaging and Characterization Core Lab, King Abdullah University of Science and Technology, Thuwal 23955-6900, Kingdom of Saudi Arabia*

[†] *These authors contributed equally to this work.*

**Corresponding authors: osman.bakr@kaust.edu.sa and omar.abdelsaboor@kaust.edu.sa*

Methods

Materials

Tetrakis(acetonitrile)copper(I) tetrafluoroborate ($[Cu(CH_3CN)_4]BF_4$, 97%) (dimethylsulfide)gold(I) chloride $(CH_3)_2SAuCl$, (R)-4-benzylthiazolidine-2-thione (R-BTT, ≥97%), (S)-4-benzylthiazolidine-2-thione (S-BTT, ≥95%), poly(sodium 4-styrenesulfonate) (PSS:Na), and lithium fluoride (LiF, ≥99.99%) were purchased from Sigma-Aldrich. HPLC-grade solvents acetonitrile (CH_3CN), methanol (MeOH), dichloromethane (CH_2Cl_2), triethylamine (Et_3N), acetone, isopropanol, and ethanol were purchased from VWR Chemicals. Poly(3,4-ethylenedioxythiophene)-poly(styrenesulfonate) (PEDOT:PSS, AI4083) was purchased from Heraeus. 1,3,5-Tris(1-phenyl-1Hbenzimidazol-2-yl)benzene (TPBi, >99.8%) was purchased from Lumtec. 4,4',4''-Tris(carbazol-9-yl)triphenylamine (TCTA, >99%) was purchased from Xi'an Polymer Light Tech. Corp. All chemicals were used directly without further purification.

Synthesis of Cu_2Au_2(R/S-BTT)$_4$.

$[Cu(CH_3CN)_4]BF_4$ (1.3 g, 4.13 mmol) was dissolved in 20 mL CH_3CN. Add R/S-BTT (1.0 g, 4.78 mmol), 20 mL MeOH, 10 mL CH_2Cl_2, and $(CH_3)_2SAuCl$ (0.8 g, 2.72 mmol) into the solution successively under vigorous stirring for 5 min. After that, the solution was centrifuged. 1 mL Et_3N was rapidly added into the supernatant, yielding a crude product of Cu_2Au_2(R/S-BTT)$_4$ in yellow-green precipitate. The precipitate was washed with 30 mL CH_3CN twice. Dissolve the precipitate into CH_2Cl_2 to get a saturated solution and then add CH_3CN into the solution, keeping the ratio of CH_2Cl_2:CH_3CN as 5:1. The solution was kept in an uncapped vial at room temperature for five days, giving pale-green crystals (~ 1.5 g).

Precursors and films preparation.

The precursors were prepared by dissolving Cu_2Au_2(R/S-BTT)$_4$ into CH_2Cl_2 at a concentration of 15 mg mL^{-1}, filtered with 0.45μm PTFE before use. As for those incorporating TCTA, different weight ratios of TCTA were mixed with Cu_2Au_2(R/S-BTT)$_4$ before dissolving into CH_2Cl_2, while maintaining a total solute concentration of 15 mg mL^{-1}. To prepare the films, 80 μL precursor was spin-coated on the substrates at 5000 rpm. for 60 s with an acceleration of 5000 rpm s^{-1} and annealed at 40 °C for 2 min.

CP-LED devices fabrication.

Glass substrates coated with patterned ITO (15 Ω sq^{-1}) were first cleaned with detergent, then ultrasonically cleaned with deionized water, acetone, isopropanol, and ethanol for 20 min in sequence. The ITO-glass substrates were dried with nitrogen flow and treated with ultraviolet ozone for 15 min before use. 150 μL modified PEDOT:PSS (m-PEDOT:PSS) was coated on the ITO-glass at 9000 rpm. for 40 s and annealed at 150 °C for 15 min. (m-PEDOT:PSS was synthesized according to the literature[1] and filtered with 0.45 μm PTFE before use.) After annealing, the substrates were quickly transferred to a nitrogen-filled glovebox. 80 μL precursors were spin-coated on the substrates at 5000 rpm. for 60 s with an acceleration of 5000 rpm s^{-1} and annealed at 40 °C for 2 min. Finally, the substrates were transferred into a high-vacuum thermal evaporator, where TPBi (35 nm), LiF (1 nm), and Al (110 nm) were deposited layer by layer at a pressure under 10^{-6} Pa. The device active area was 2.5 × 4 mm^2 as defined by the overlapping area of the ITO and Al electrodes.

Single-crystal XRD measurements.

Single-crystal X-ray diffraction data were collected using a Bruker D8 Venture diffractometer with a SMART APEX2 area detector (Mo Kα, λ = 0.71073 Å) at 120 K. Data processing was performed using Bruker's APEX3 software. Data integration and reduction were performed using SaintPlus.

Absorption corrections were performed by the multi-scan method implemented in SADABS-2016/2. Space groups were determined using XPREP implemented in APEX32. The structures were solved with the ShelXT[2] structure solution program using intrinsic phasing and refined with the ShelXL-2015[3] refinement package by least squares minimization using Olex2[4]. All non-hydrogen atoms were in difference-Fourier maps and were then refined anisotropically. All hydrogen atoms were assigned isotropic displacement coefficients U(H) = 1.2U or 1.5U, and their coordinates were allowed to ride on their respective atoms.

The X-ray crystallographic data for $Cu_2Au_2(R\text{-}BTT)_4$[5] (deposition number: 2205603) and $Cu_2Au_2(S\text{-}BTT)_4$ (deposition number: 2288770) have been deposited into the Cambridge Crystallographic Data Centre (CCDC). The X-ray crystallographic data were deposited into the Cambridge Crystallographic Data Centre (CCDC). These data can be obtained free of charge from the CCDC via www.ccdc.cam.ac.uk.

Photoluminescence and absorption characterizations.

An Edinburgh Fluorescence Spectrometer (FLS 920) was used for PL, PLE, and 3D-EEM spectra measurements. The PLQYs and PL spectra were measured with an integrating sphere and a monochromatized xenon lamp as the excitation source. The 3D-EEM spectra were measured by a step-excitation wavelength of 5 nm. The PL intensity evolutions were recorded using a Horiba Fluromax-4 spectrofluorometer. Ultraviolet-visible absorption was measured using a spectrometer (JAZ, Ocean Optics) and an ultraviolet-visible-infrared light source (DH-2000-BAL, Ocean Optics).

Femtosecond Transient absorption (fs-TA) measurements.

The transient spectra and their kinetics were obtained through fs-TA spectroscopy in a Helios spectrometer (Ultrafast Systems). For this purpose, the samples were excited with 365-nm pulses

obtained from an optical parametric amplifier pumped by an amplified Ti:Sapphire laser (800 nm, 150 fs, 1 kHz, Astrella-Coherent). The probe pulses (white light) were generated by passing another fraction of the 800-nm beam in mechanical delay stage and later through a 2-mm thick CaF2 crystal for the visible range. The white light was split into two beams (signal and reference). The excitation pump pulses were spatially overlapped with the probe pulses on the samples after passing through a synchronized mechanical chopper (500 Hz) which blocked alternative pump pulses. The obtained signal was sent to the detector through an optical fiber. The absorption change (ΔA) was measured with respect to the time delay and wavelength (λ). The kinetic traces were fitted using the Lavenberg-Marquart algorithm as implemented in Ultrafast System software.

Time-resolved photoluminescence (TRPL) decay measurements.

The TRPL was measured through the time-Correlated Single-Photon Counting measurements (TCSPC) that were performed in a Halcyone setup (Ultrafast Systems), the excitation wavelength was selected to be 365 nm using a parametric optical amplifier (Newport, Spectra-Physics) that was pumped with an Astrella femtosecond pulsed laser (800 nm, 150 fs, 1 kHz, Coherent). Photoluminescence at 540 nm was collected, and recollimated by a pair of parabolic mirrors passed through a longpass filter (490 nm, Newport) and finally focused on an optical fiber coupled to a monochromator and a PMT detector. The energy at each excitation wavelength was set constant with the help of a pair of variable neutral density filters (Thorlabs) to ensure that less than 1 % of excitation events resulted in a detected photon. TCSPC histograms were fitted using the Lavenberg-Marquart algorithm as implemented in Ultrafast System software. The overall time resolution for the system was better than 120 ps.

Transmission electron microscopy (TEM) measurements.

Cross-section TEM samples were prepared by the focused ion beam (Helios G4 UX). TEM images and the corresponding EDS mappings were acquired by using an aberration-corrected FEI (Themis Z) at 300 kV.

Scanning electron microscopy (SEM) and atomic force microscopy (AFM) measurements.

SEM images were acquired using a Helios G4 UX dual beam scanning electron microscopy. AFM using a Digital Instrument Multimode AFM (Veeco Metrology Group) equipped with specific AFM tips (Model: OLTESPA, nominal spring constant 0.5-4.4 N/m). The AFM tips were coated with 50 ± 10 nm Al on back sides.

Ultraviolet photoelectron spectroscopy (UPS) measurements.

UPS studies were carried out in a Kratos Axis Ultra DLD spectrometer equipped with a monochromatic Al $K\alpha$ X-ray source (hν = 1486.6 eV) operating at 150 W, a multi-channel plate and delay line detector under a vacuum of ~10^{-9} mbar. All spectra were recorded using an aperture slot of 300 x 700 μm. Survey spectra were collected using a pass energy of 160 eV and a step size of 1 eV. A pass energy of 20 eV and a step size of 0.1 eV were used for the high-resolution spectra. Samples were mounted in floating mode to avoid differential charging.

Density functional theory (DFT) calculation.

The geometry of Cu_2Au_2(R/S-BTT)$_4$ were optimized using the hybrid functional B3LYP as implemented in Gaussian09 (version D.01). The LANL2DZ basis set was used for Cu and Au atoms, and the 631G(d) basis set was used to describe H, C, N, and S atoms. The same hybrid functional and basis sets were used to optimize the geometry of Cu_2Au_2(R/S-BTT)$_4$ in the excited state using the time-dependent DFT (TDDFT) method. Based on the optimized geometries, the dipole moments and charge densities for the frontier orbitals of Cu_2Au_2(R/S-BTT)$_4$ were obtained.

Performance evaluation of LED.

The LED devices were measured in a nitrogen-filled glovebox at room temperature. The current density-voltage curves were recorded using a Keithley 2400 source meter. The EL and absolute radiation flux for calculating luminance, EQE, CE, PE, and CIE were measured using a commercialized system (LQ-100X, Enlitech) that was equipped with an integrating sphere and a photomultiplier tube (PMT). A commercial inorganic LED (Enlitech, RR2110501) to calibrate and cross-check the LED measurements.

Circular dichroism (CD) spectra and circularly polarized luminescence (CPL) measurements.

CD and CPL spectra were recorded on a Chirascan V100 spectropolarimeter and a JASCO CPL-300 spectrometer, respectively. The CPPL was excited at 365 nm and the CPEL was obtained under a constant driving voltage at 5.5 V.

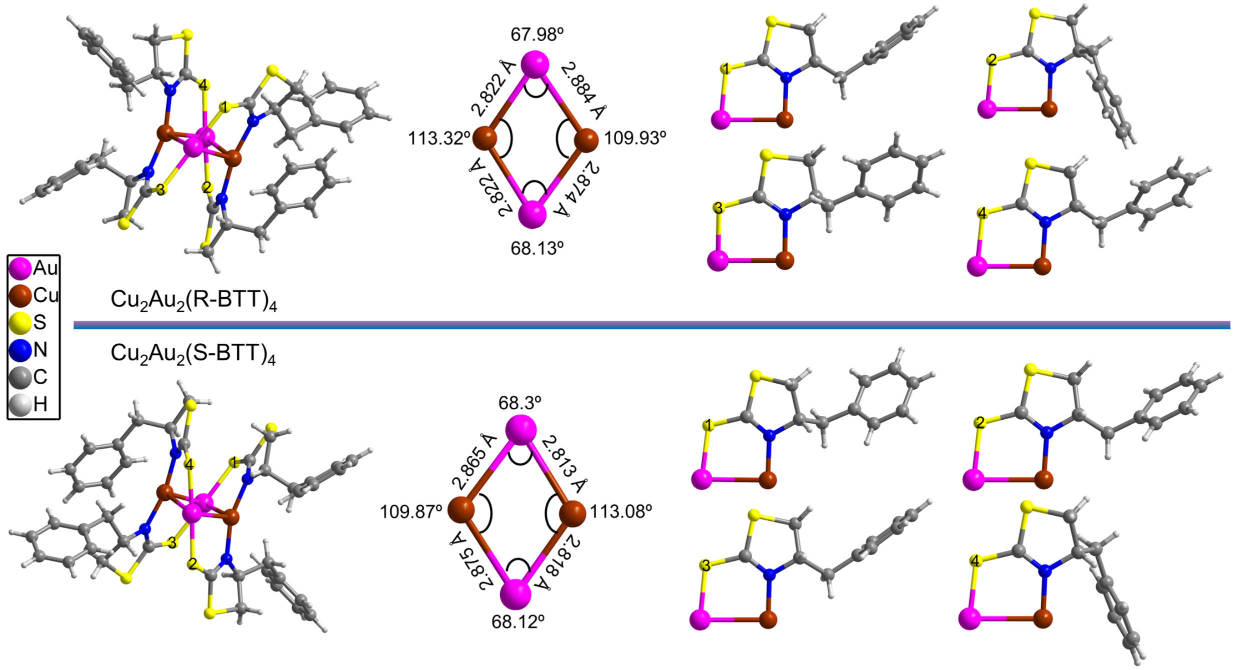

Figure S1. Ball-stick model of Cu_2Au_2(R/S-BTT)$_4$ structure, quasi-rhombic geometry of the tetranuclear Cu-Au structure, and the spatial orientation of the benzyl groups.

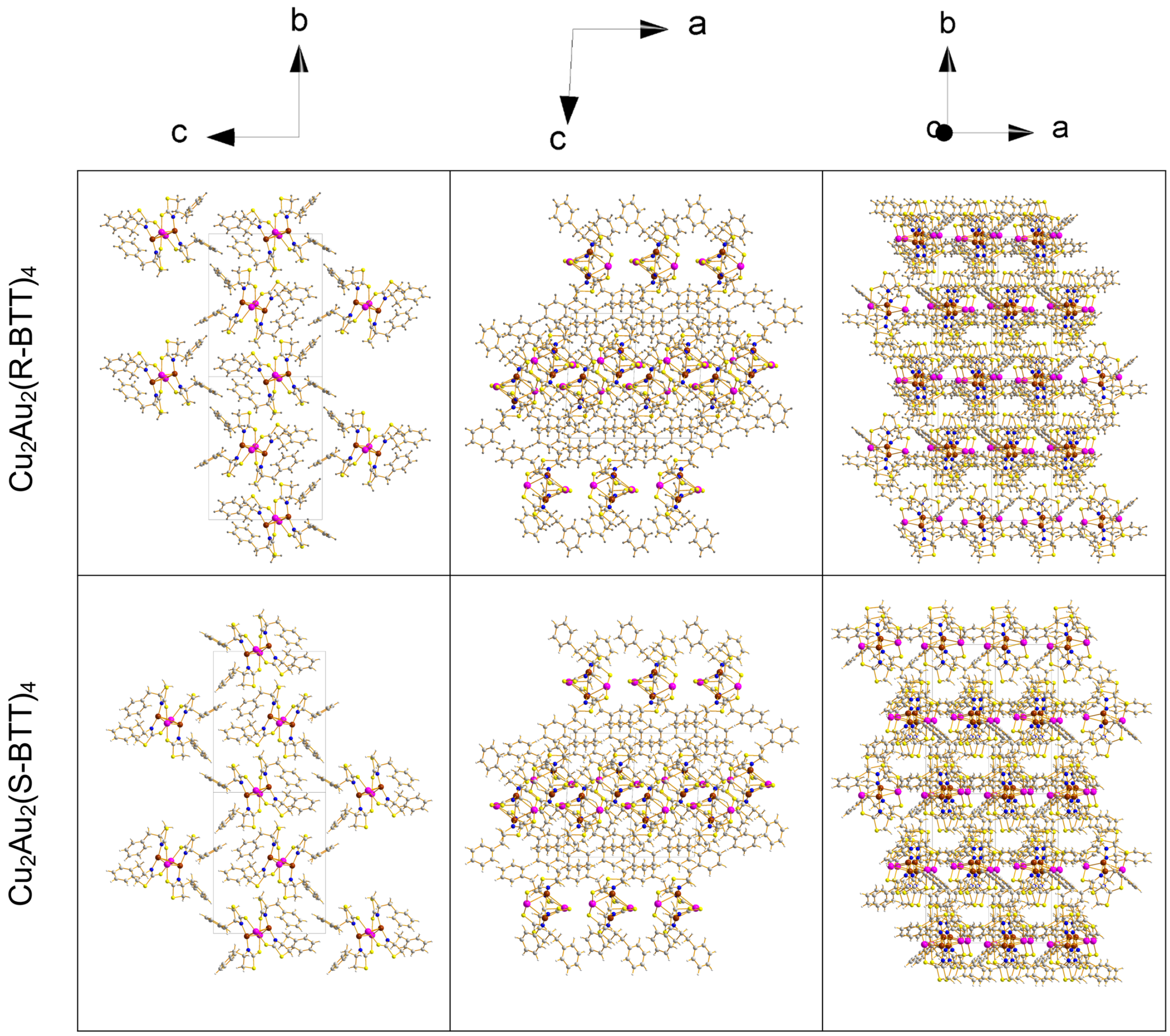

Figure S2. The packing model of Cu_2Au_2(R/S-BTT)$_4$ in crystalline solid state.

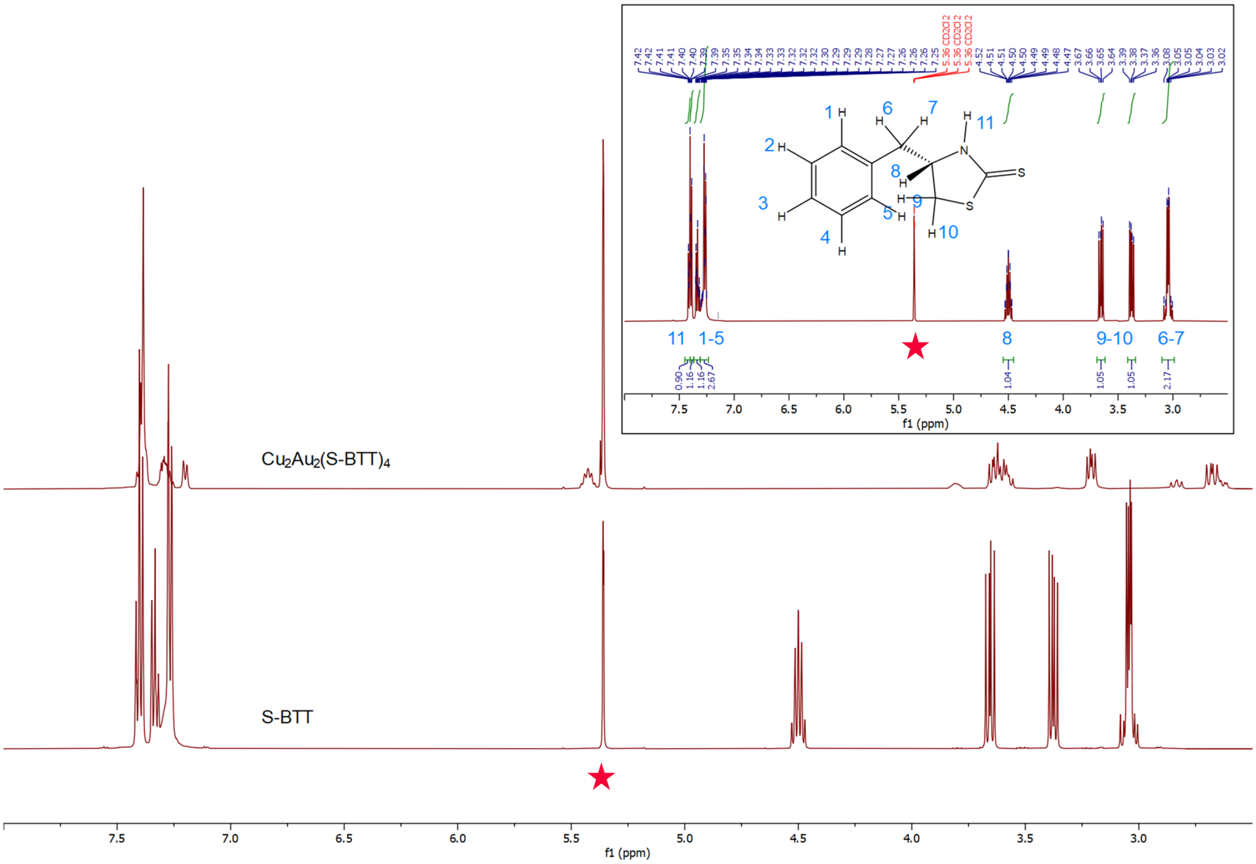

Figure S3. ^{1}H NMR (CD_2Cl_2, 600 MHz) spectra of Cu_2Au_2(S-BTT)$_4$ and S-BTT. Inset: the structural formula and ^{1}H NMR spectrum of S-BTT. The red star denotes the solvent residual peak.

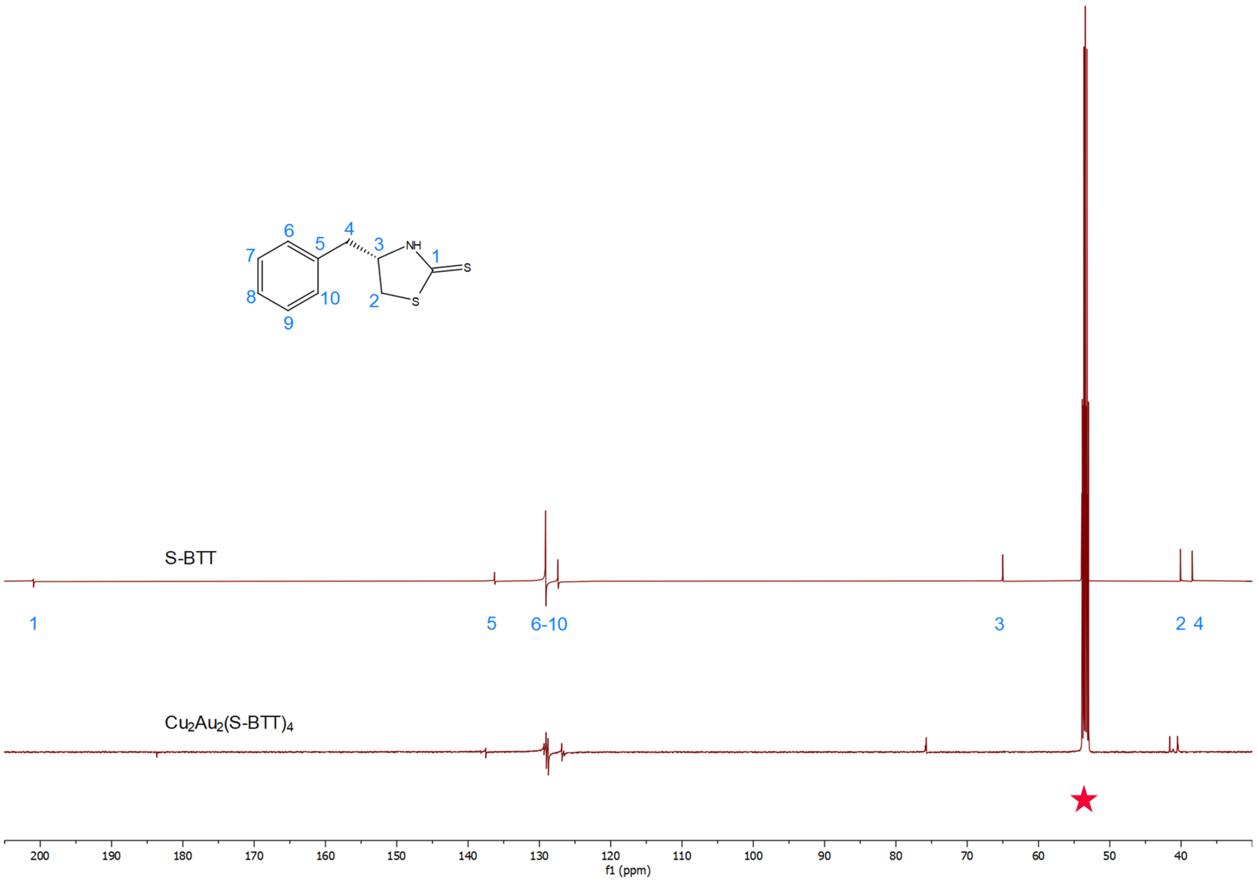

Figure S4. ^{13}C NMR (CD_2Cl_2, 600 MHz) spectra of $Cu_2Au_2(S\text{-}BTT)_4$ and S-BTT. Inset: the structural formula of S-BTT. The red star denotes the solvent residual peak.

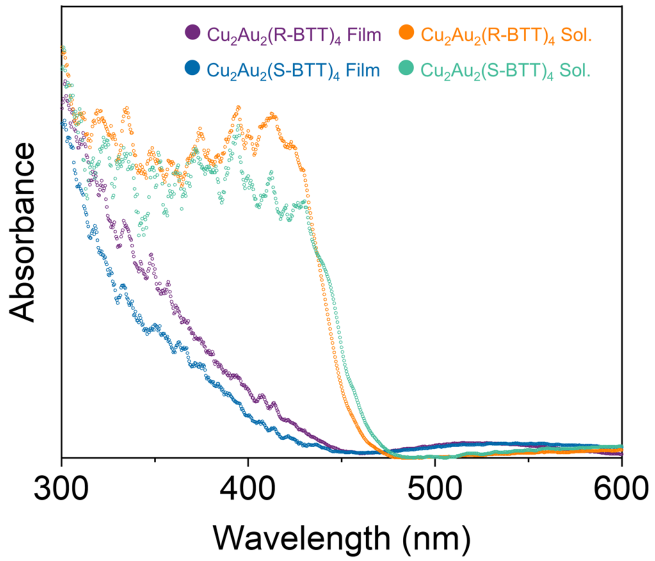

Figure S5| Ultraviolet-visible absorption spectra of Cu_2Au_2(R/S-BTT)$_4$ in film and solution states.

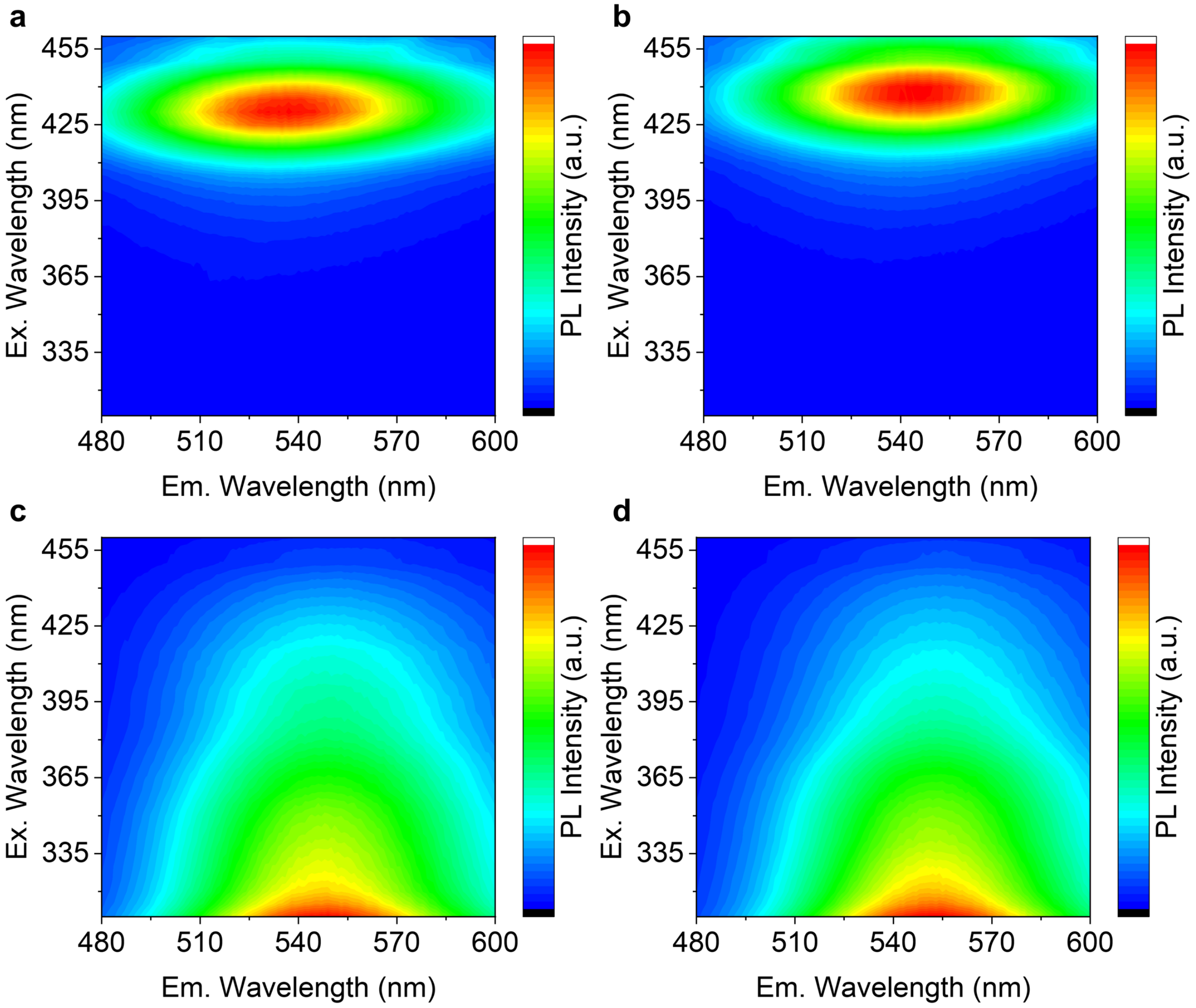

Figure S6| Three-dimensional excitation-emission matrix (3D-EEM) luminance spectra of Cu_2Au_2(R-BTT)$_4$ (**a**) solution and (**b**) film, and Cu_2Au_2(S-BTT)$_4$ (**c**) solution and (**d**) film.

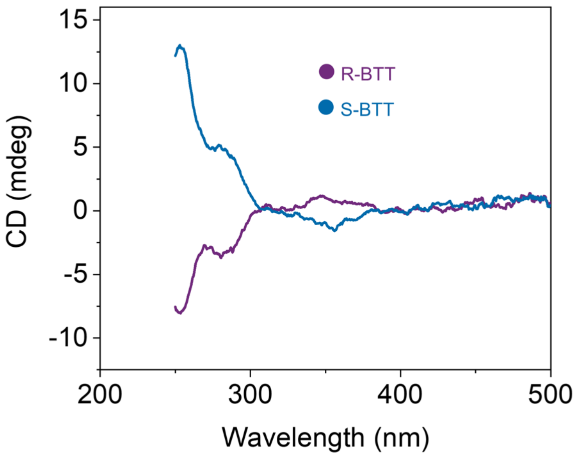

Figure S7| Circular dichroism (CD) spectra of the ligands of R/S-BTT.

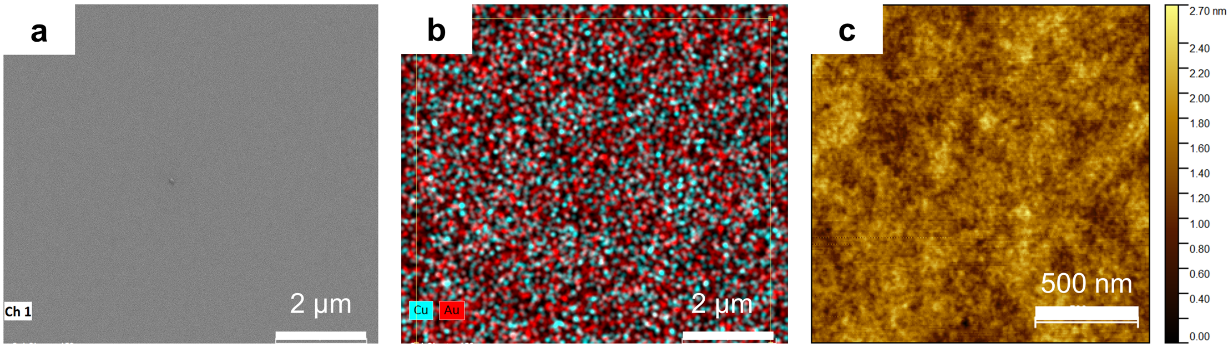

Figure S8| Top surface morphology of cluster film. **a,** Scanning electron microscopy (SEM) image, **b,** energy dispersive spectrum (EDS), and **c,** atomic force microscopy (AFM) image of Cu_2Au_2(R-BTT)$_4$ films.

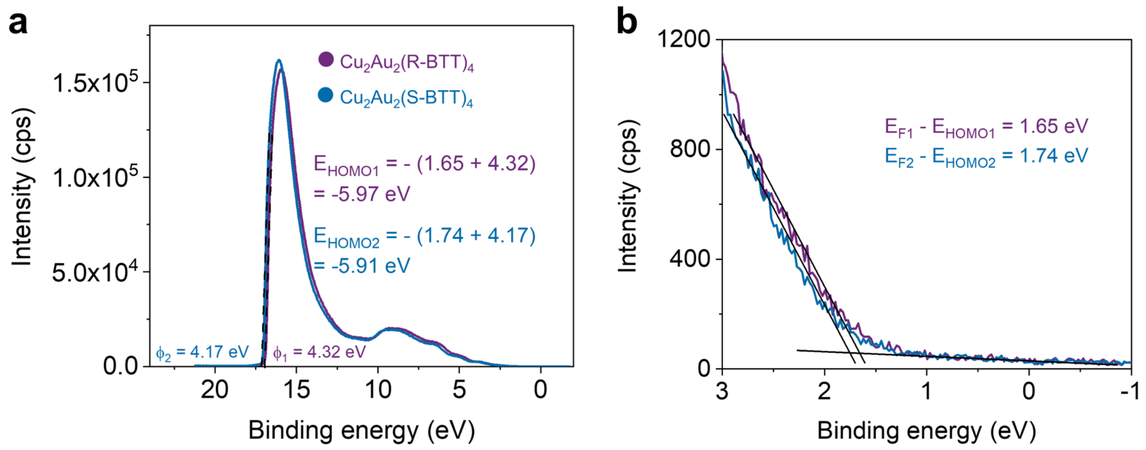

Figure S9| Ultraviolet photoelectron spectroscopy (UPS) spectra of Cu_2Au_2(R/S-BTT). **a,** Full spectrum and **b,** Fermi edge.

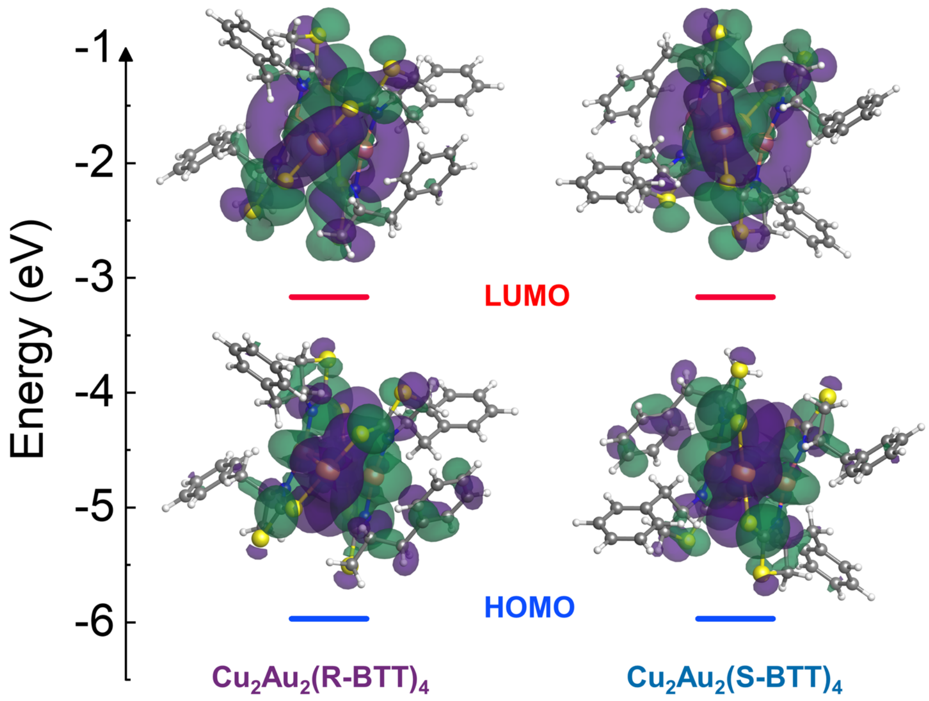

Figure S10| Electronic charge densities and energy levels for the highest occupied molecular orbital (HOMO) and the **lowest** unoccupied molecular orbital (LUMO) of Cu_2Au_2(R/S-BTT).

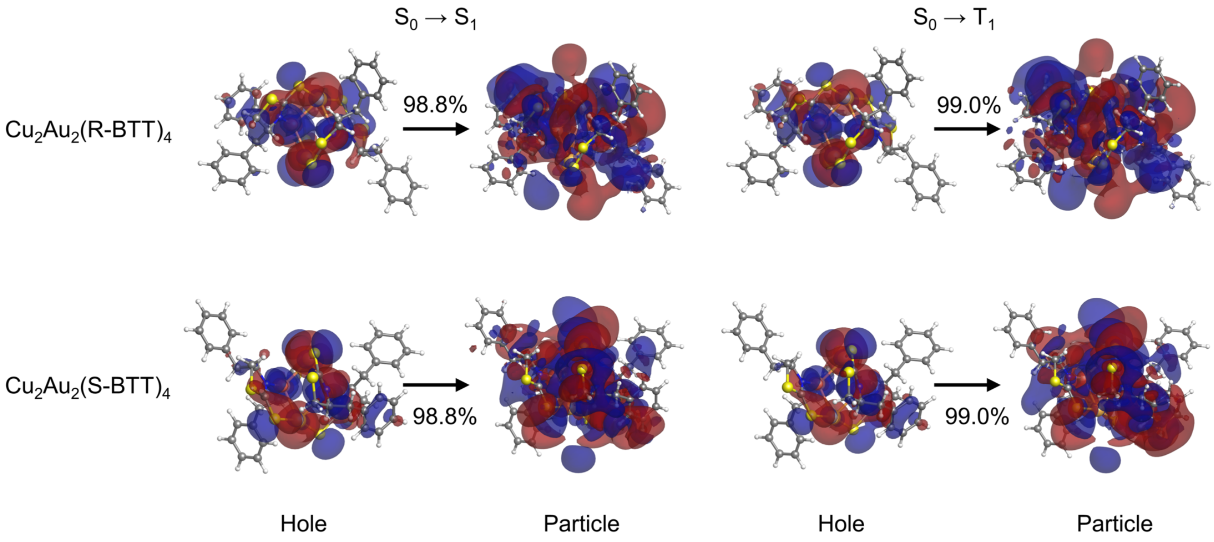

Figure S11| Contours of the highest occupied ("hole") and the lowest unoccupied ("particle") natural transition orbitals (NTOs) for the singlet (S_1) and triplet (T_1) states of Cu_2Au_2(R/S-BTT)$_4$.

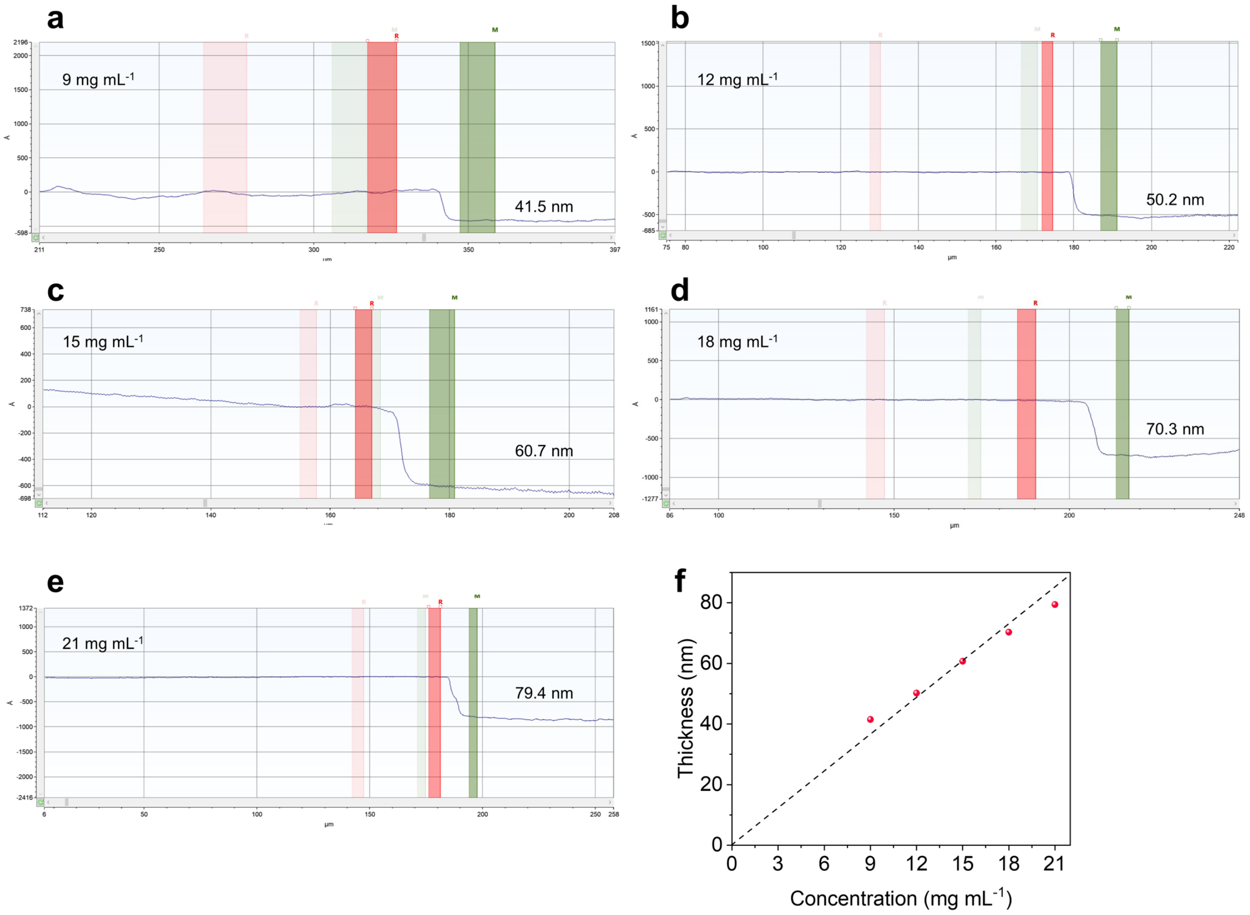

Figure S12| Variation in Cu_2Au_2(R-BTT)$_4$ film thickness with different precursor concentrations. **a-e,** Screenshots illustrate the measured film thickness (obtained using a Bruker DektakXT profilometer). **f,** Film thickness is plotted as a function of the precursor concentration.

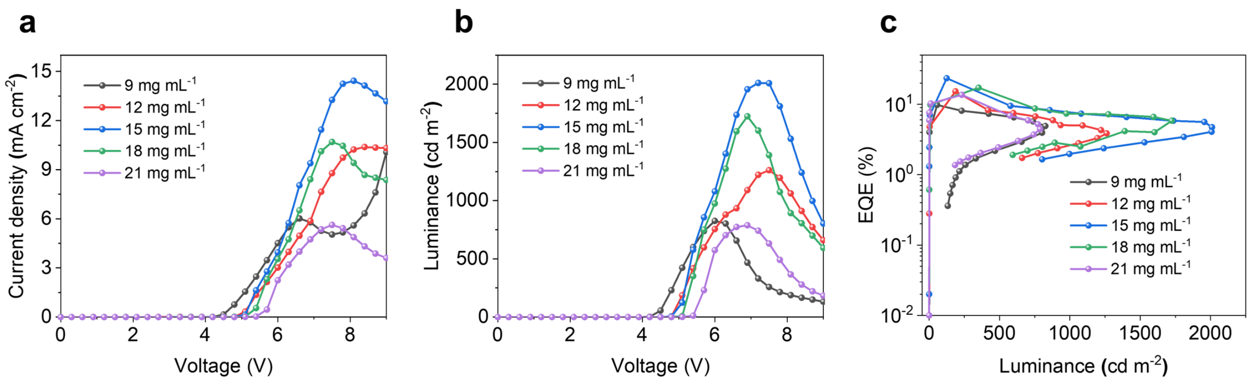

Figure S13| Performances of CP-LEDs based on different precursor concentrations. **a,** Current density-voltage, **b,** luminance-voltage, and **c,** EQE-luminance curves for the CP-LEDs prepared with different precursor concentrations.

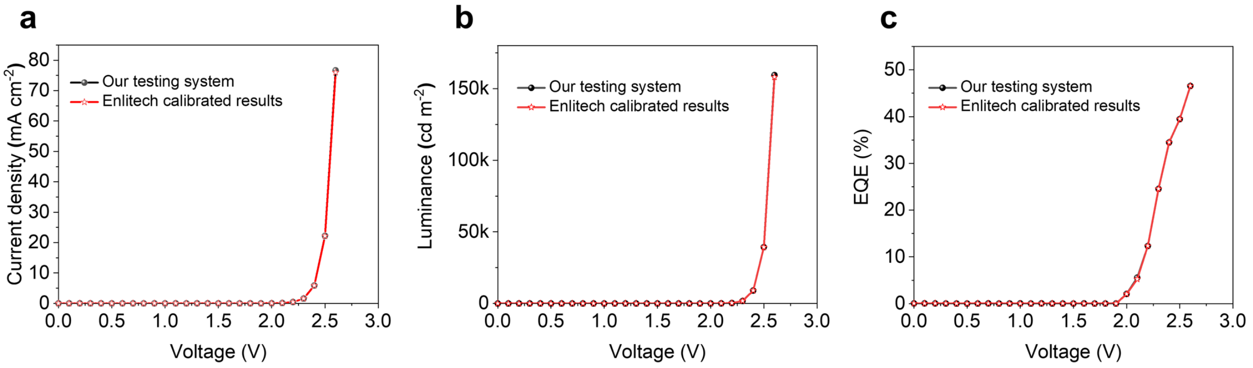

Figure S14| Calibration of the LED testing system. **a,** Current density-voltage, **b,** luminance-voltage, and **c,** EQE-voltage curves comparing results obtained from our testing system with those from the instrument's manufacturer, Enlitech, for a commercially available inorganic LED.

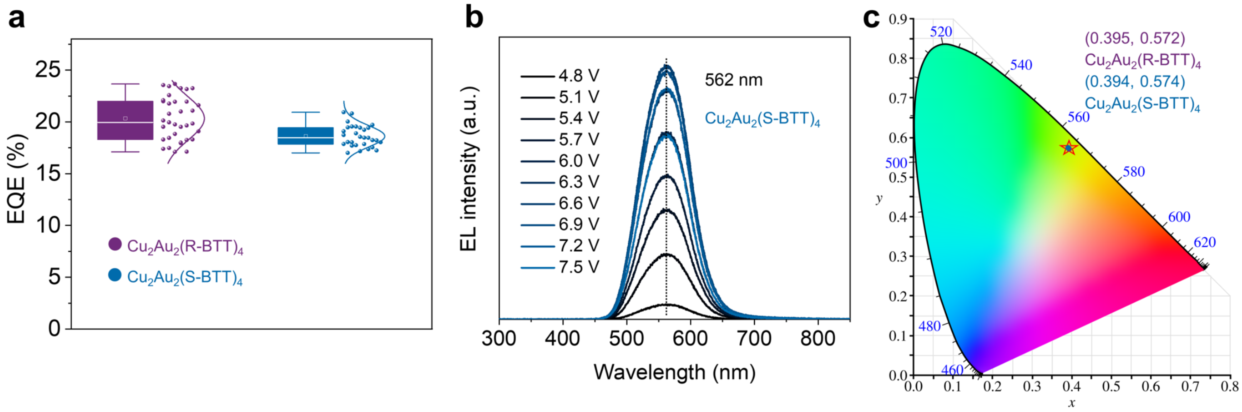

Figure S15| Performances of CP-LED device based on Cu_2Au_2(R/S-BTT)$_4$. **a,** Statical maximum external quantum efficiencies (EQEs). **b,** Electroluminescence (EL) spectra of CP-LEDs based on Cu_2Au_2(S-BTT)$_4$. **c,** CIE coordinates.

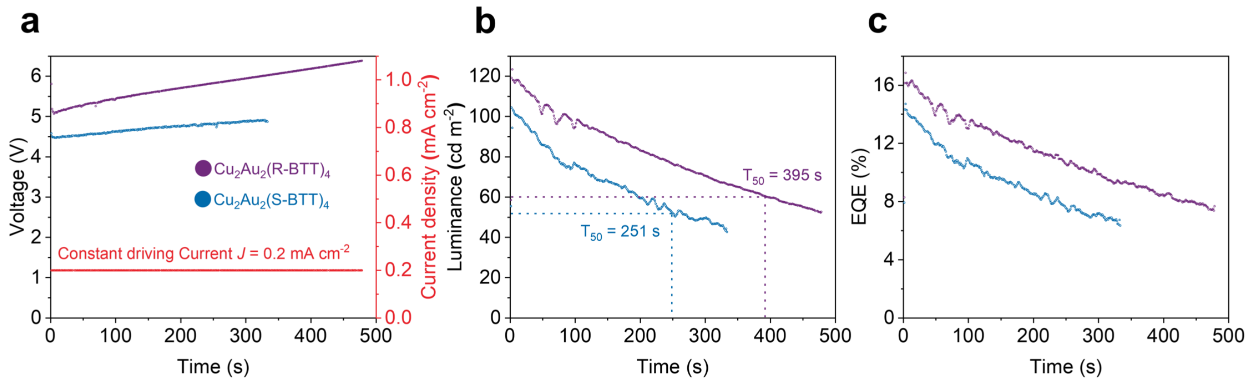

Figure S16| Operational lifetime performances of the CP-LEDs based on Cu_2Au_2(R-BTT)$_4$ and Cu_2Au_2(S-BTT)$_4$. **a,** Driving current density and voltage evolution curves of the CP-LED devices. **b,** Luminance and **c,** EQE evolution curves of the CP-LED devices under a constant current driving.

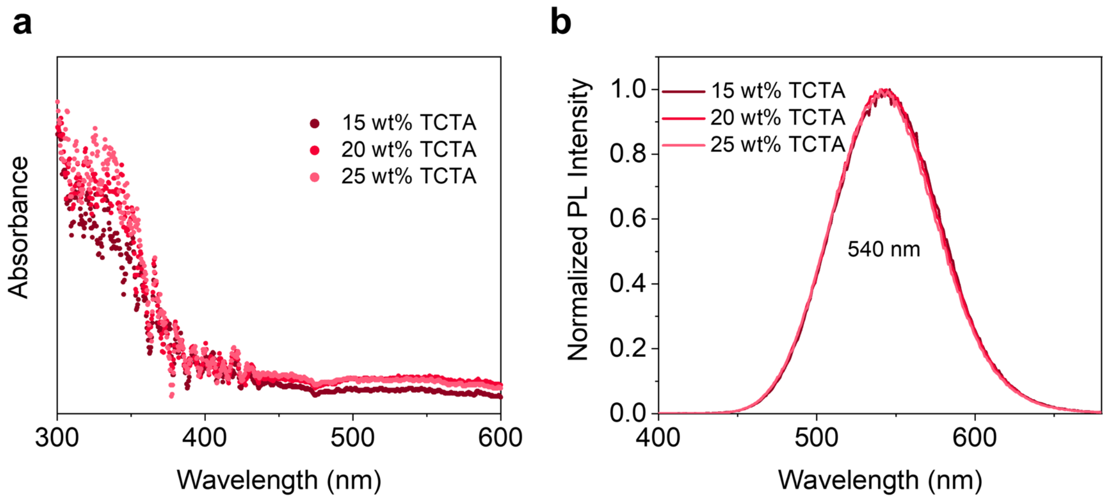

Figure S17| a, Absorption and **b,** PL spectra of Cu_2Au_2(R-BTT)$_4$ films with different weight ratios of TCTA incorporation.

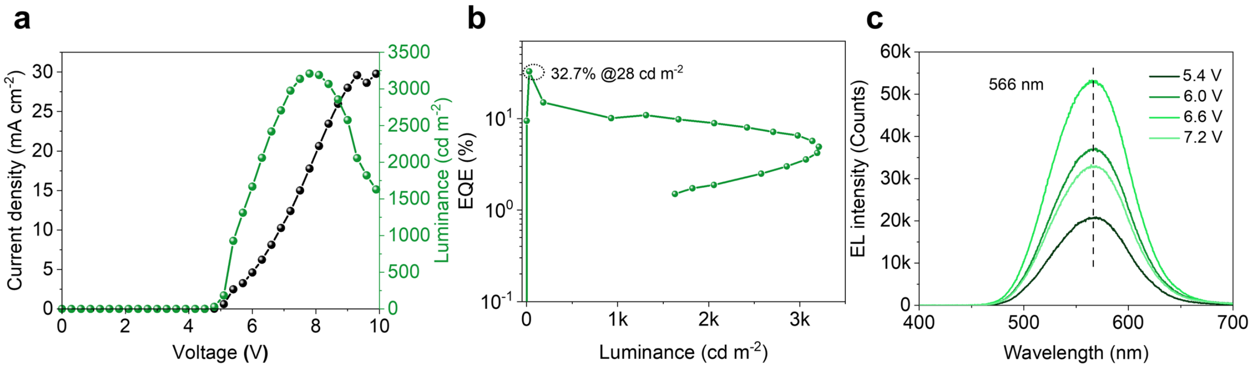

Figure S18| Device performances of CP-LEDs based on $Cu_2Au_2(S\text{-}BTT)_4$ with 20 wt% TCTA incorporation. **a,** Luminance-voltage-current density curves. **b,** EQE-luminance curve. **c,** EL spectra under different driving voltage.

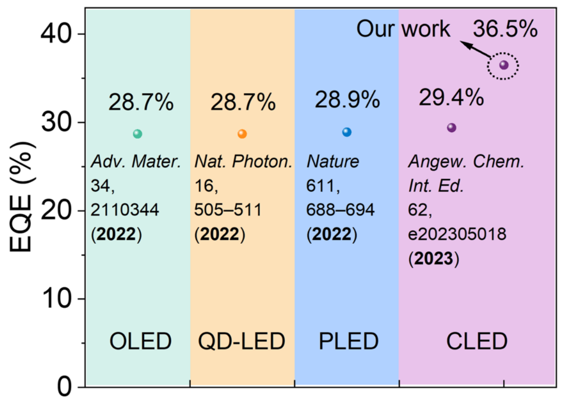

Figure S19| Summary of the maximum EQEs of state-of-the-art solution-processed LED technologies.

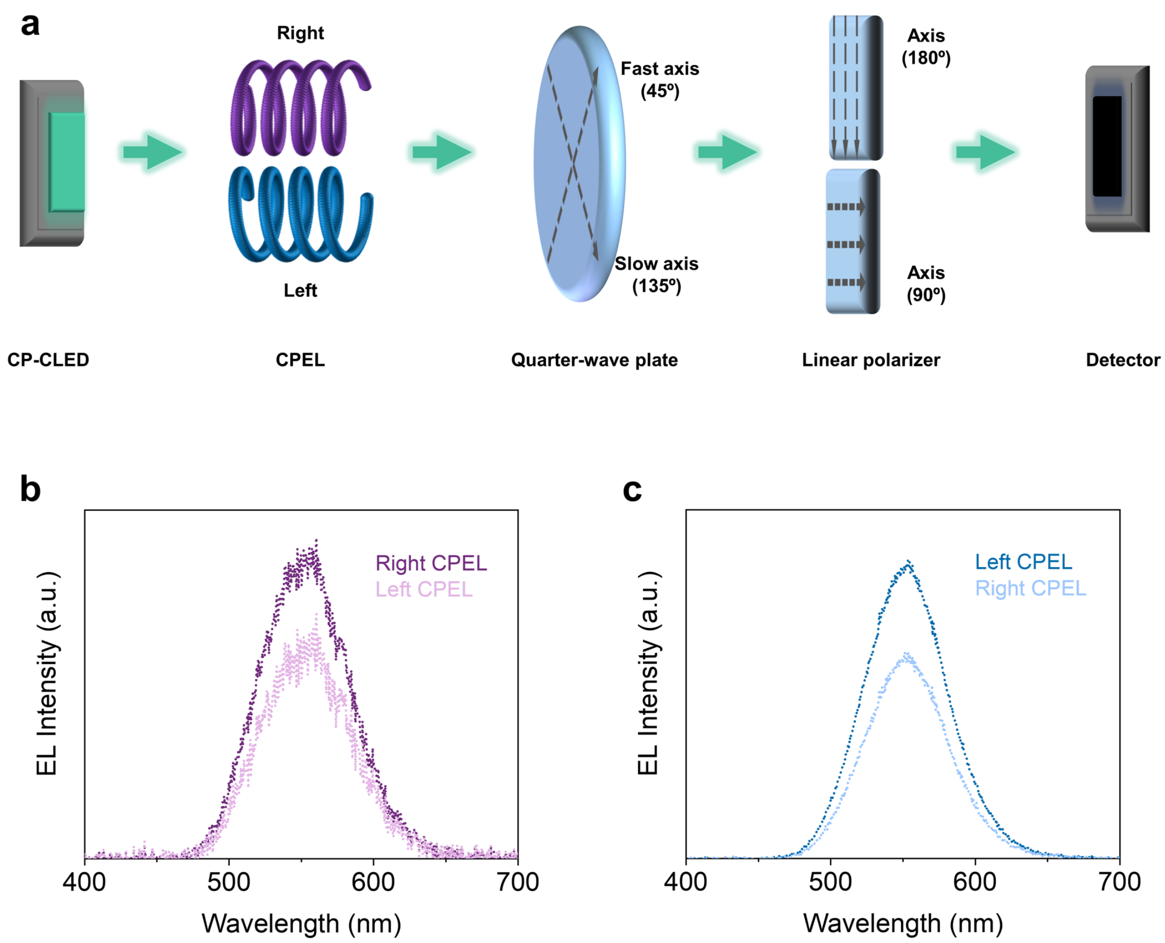

Figure S20| a, The schematic diagram of the CPEL spectra detection. CPEL spectra of the CP-LEDs based on (**b**) Cu_2Au_2(R-BTT)$_4$ with 20 wt% TCTA and (**c**) Cu_2Au_2(S-BTT)$_4$ with 20 wt% TCTA.

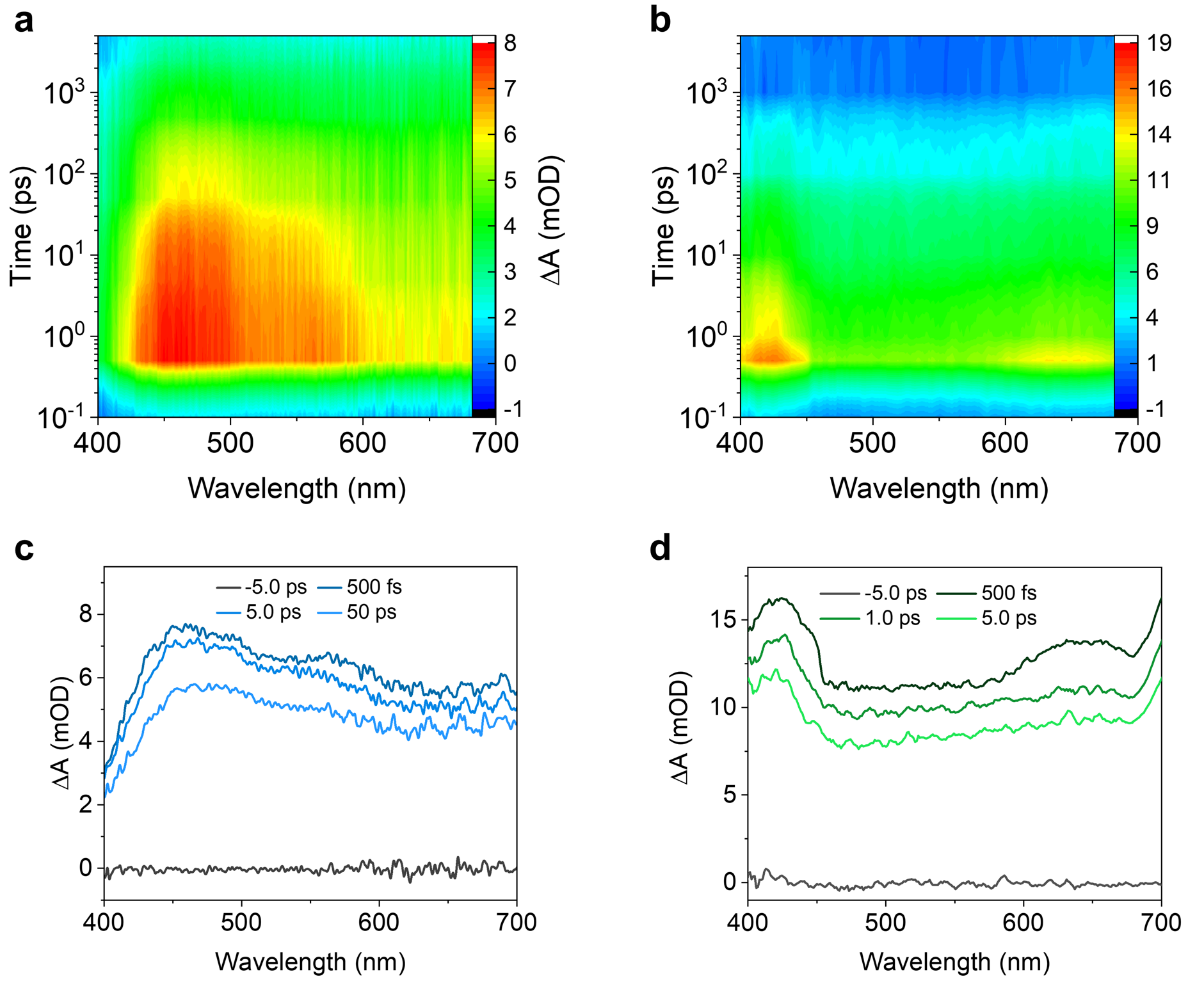

Figure S21| Two-dimensional (2D) pseudo-color TA spectra of **a,** pure Cu_2Au_2(S-BTT)$_4$ and **b,** Cu_2Au_2(S-BTT)$_4$ with 20 wt% TCTA. TA spectra at different delay times of **c,** pure Cu_2Au_2(S-BTT)$_4$ and **d,** Cu_2Au_2(S-BTT)$_4$ with 20 wt% TCTA.

Table S1| Single-crystal X-ray crystallography data for Cu_2Au_2(R-BTT)$_4$ and Cu_2Au_2(S-BTT)$_4$.

	Cu_2Au_2(R-BTT)$_4$[#]	Cu_2Au_2(S-BTT)$_4$
Formula	$C_{41}H_{42}Au_2Cl_2Cu_2N_4S_8$	$C_{41}H_{42}Au_2Cl_2Cu_2N_4S_8$
Formula weight	1439.18	1439.18
Temperature/K	120	99.99
Crystal system	monoclinic	monoclinic
Space group	$P2_1$	$P2_1$
a/Å	8.0774(10)	8.0527(10)
b/Å	19.128(2)	19.109(3)
c/Å	15.229(2)	15.221(2)
α/°	90	90
β/°	93.229(6)	93.220(6)
γ/°	90	90
Volume/Å^3	2349.3(5)	2338.5(6)
Z	2	2
ρ_{calc}g/cm^3	2.035	2.044
μ/mm^{-1}	7.623	7.659
F(000)	1388.0	1388.0
Crystal size/mm^3	0.23 × 0.15 × 0.03	0.21 × 0.12 × 0.03
Radiation	Mo Kα (λ = 0.71073)	Mo Kα (λ = 0.71073)
2θ range for data collection/°	5.052 to 66.318	5.066 to 66.238
Index ranges	$-11 \leqslant h \leqslant 12, -29 \leqslant k \leqslant 29, -21 \leqslant l \leqslant 23$	$-10 \leqslant h \leqslant 12, -29 \leqslant k \leqslant 29, -23 \leqslant l \leqslant 22$
Reflections collected	40433	52676
Independent reflections	17242 [R_{int} = 0.0325, R_{sigma} = 0.0662]	17237 [R_{int} = 0.0398, R_{sigma} = 0.0571]
Data/restraints/parameters	17242/1/532	17237/1/532
Goodness-of-fit on F^2	0.854	0.871
Final R indexes [I>=2σ (I)]	R_1 = 0.0265, wR_2 = 0.0542	R_1 = 0.0241, wR_2 = 0.0496
Final R indexes [all data]	R_1 = 0.0303, wR_2 = 0.0570	R_1 = 0.0263, wR_2 = 0.0508
Largest diff. peak/hole / e Å^{-3}	1.15/−1.53	1.23/−1.40
Flack parameter	0.029(3)	0.038(3)

[#]According to our previous literature[5].

Table S2| Performance summary of CP-LEDs prepared with different precursor concentrations.

Concentration (mg mL^{-1})	Thickness (nm)	Turn-on Voltage (V)	Luminance (cd m^{-2})	EQE (%)
9	41.5	3.9 – 4.2	826	9.8
12	50.2	4.5 – 4.8	1,261	15.3
15	60.7	4.5 – 4.8	2,010	23.5
18	70.3	5.1 – 5.4	1,724	17.4
21	79.4	5.4 – 5.7	788	13.6

Table S3| Summary of the performances of the optimal LED devices.

Emitting layer	Turn-on voltage (V)	Luminance (cd m^{-2})	PL peak (nm)	EL peak (nm)	EQE (%)	CE (cd A^{-1})	PE (lm W^{-1})
Cu_2Au_2(R-BTT)$_4$	4.5 – 4.8	2,010	540	564	23.5	87.4	53.9
Cu_2Au_2(S-BTT)$_4$	4.5 – 4.8	1,690	540	562	20.8	77.7	50.8
Cu_2Au_2(R-BTT)$_4$ /TCTA (20 wt%)	4.5 – 4.8	3,205	540	569	36.5	134.1	82.6
Cu_2Au_2(S-BTT)$_4$ /TCTA (20 wt%)	4.5 – 4.8	3,197	540	569	32.7	119.6	78.3

Table S4| Summary of the performance of the LEDs using clusters or cluster complexes as parts of the emitting center.

	Emitting center	ϕ_{PL} (%)	EL peak (nm)	Luminance (cd m^{-2})	EQE (%)	Ref.
Cluster complex doping	*m*CP:OXD-7:Au_4Ag_2 complex (53:27:20 wt%)	53.5	539	17,160	7.0	*Adv. Funct. Mater.* **2015**, 25, 3033
	TCTA:OXD-7:Ag_6Cu complex (57:28:15 wt%)	78	573	3,000	13.9	*J. Mater. Chem. C.* **2016**, 4, 1787
	*m*CP:OXD-7:$PtAu_3$ complex (48.5:48.5:3 wt%)	76.8	588	19,308	18.1	*J. Mater. Chem. C.* **2018**, 6, 8966
	*m*CP:OXD-7:$PtAu_3$ complex (47.5:47.5:5 wt%)	87.3	556	6,539	16.6	*J. Mater. Chem. C.* **2019**, 7, 2604
	2,6DCZPPY:Ag_8Au_{10} complex (95:5 wt%)	91	567	14,859	15.7	*ACS Appl. Mater. Interface* **2020**, 12, 57264
	TCTA:OXD-7:Ag_3Cu_5 complex (47.5:47.5:5 wt%)	75	585	8,554	14.7	*J. Mater. Chem. C.* **2021**, 9, 5528
Cluster complex	[Cu_6I_6(ppda)$_2$] complex	15	564	100	0.31	*Dalton Trans.* **2020**, 49, 5859
Cluster doping	*m*CP:[DBFDP]$_2Cu_4I_4$ (90:10 wt%)	5	550	1,500	0.73	*Chem. Mater.* **2017**, 29, 6606
	CzSi:[DtBCzDBFDP]$_2Cu_4I_4$ (90:10 wt%)	65	492	6,772	7.9	*Sci. Adv.* **2019**, 5, eaav9857
	TPBi:[O(Audppy)$_3$]BF_4 (80:20 wt%)	-	436	8,313	2.42	*Angew. Chem. Int. Ed.* **2022**, 61, e202213826
	BCPO:DMACDBFDPCu_4I_4 (60:40 wt%)	81	500	4,440	19.5	*J. Am. Chem. Soc.* **2022**, 144, 6551
	BCPO:[tBCzDBFDP]$_2Cu_4I_4$ (60:40 wt%)	68	495	1,000	12.2	*Research* **2022**, 2022:0005
	*m*CP:R/S-Ag_6(PTLT)$_6$ (80:20 wt%)	71	578	3,906	10	*Nano Res.* **2023**, 10.1007/s12274-022-5285-3
	CzAcSF:[DDMACDBFDP]$_2Cu_4I_4$ (80:20 wt%)	99	504	4,234	29.4	*Angew. Chem. Int. Ed.* **2023**, 62, e202305018
	**TCTA:Cu_2Au_2(R-BTT)$_4$ (20:80 wt%)**	**-**	**569**	**3,205**	**36.5**	**This work**
Cluster	$Au_{25}SG_{18}$	-	750	-	0.013	*Adv. Mater.* **2014**, 26, 1446
	Au(0)@Au(I)-thiolate	15	625	40	0.1	*Nanoscale* **2015**, 7, 9140
	Cu_2I_2(BINAP)$_2$	-	505	1,200	0.54	*J. Am. Chem. Soc.* **2021**, 143, 10860
	[DDMACDBFDP]$_2Cu_4I_4$	63	508	2,646	9.5	*Angew. Chem. Int. Ed.* **2023**, 62, e202305018
	**Cu_2Au_2(R-BTT)$_4$**	**93.7**	**564**	**2,010**	**23.5**	**This work**

Table S5| Summary of the EQE and $|g_{EL}|$ of recently reported CP-LEDs and our device.

	Materials	Processing	EQE_{max} (%)	$\|g_{EL}\|$	Ref.
Polymer	Polymer with chiral molecule dopant	Solution	< 1	0.2	*Adv. Mater.* **2013**, 25, 2624
	Chiral polymer	Solution	0.012	0.021	*J. Mater. Chem. C.* **2021**, 9, 12141
	Chiral TADF-activated polymer	Solution	22.1	0.001	*Angew. Chem. Int. Ed.* **2021**, 60, 23619
Metal complex	Platinum complex	Solution	< 1	0.38	*J. Am. Chem. Soc.* **2016**, 138, 9743
	Platinum complex	Solution	2.15	0.0011	*ACS Appl. Mater. Interfaces* **2020**, 12, 9520
	Platinum complex	Solution	14.3	0.002	*J. Am. Chem. Soc.* **2022**, 144, 2233
	Europium complex	Solution	< 1	1	*Adv. Funct. Mater.* **2017**, 27, 1603719
	Europium complex	Solution	0.22	0.51	*J. Am. Chem. Soc.* **2022**, 10, 463
	Iridium complex	Evaporation	18.8	0.0077	*Adv. Funct. Mater.* **2021**, 31, 2102898
Small organic molecule	Small chiral organic molecule	Evaporation	9.3	0.026	*Adv. Funct. Mater.* **2018**, 28, 1800051
	Small chiral organic molecule	Solution	2.3	0.023	*Adv. Mater.* **2023**, 35, 2209495
	Chiral TADF-activated small organic molecule	Evaporation	19.8	0.0023	*Angew. Chem. Int. Ed.* **2018**, 57, 2889
	Chiral TADF-activated small organic molecule	Evaporation	12.7	0.014	*Angew. Chem. Int. Ed.* **2020**, 59, 3500
	Chiral TADF-activated small organic molecule	Evaporation	23.1	0.0013	*J. Am. Chem. Soc.* **2020**, 142, 17756
	Chiral TADF-activated small organic molecule	Evaporation	28.3	0.0006	*Adv. Optical Mater.* **2021**, 9, 2100017
	Chiral TADF-activated small organic molecule	Evaporation	29.4	0.0014	*Adv. Mater.* **2021**, 33, 2100652
	Chiral TADF-activated small organic molecule	Evaporation	20	0.0031	*Angew. Chem. Int. Ed.* **2021**, 60, 8435
	Chiral TADF-activated small organic molecule	Evaporation	32.1	0.0015	*Angew. Chem. Int. Ed.* **2023**, 62, e202217045
	Chiral TADF-activated small organic molecule	Evaporation	32.6	0.002	*Adv. Mater.* **2019**, 31, 1900524
	Chiral TADF-activated small organic molecule	Solution	10.6	0.0039	*J. Am. Chem. Soc.* **2019**, 7, 14511
Exciplex	Chiral exciplex with Iridium complex dopant	Evaporation	32	0.0032	*Adv. Funct. Mater.* **2023**, 2215179
	Chiral exciplex	Evaporation	33.2	0.0028	*Adv. Mater.* **2022**, 34, 2109147

	Chiral exciplex	Evaporation	12.7	0.0073	*Adv. Optical Mater*. **2022**, 10, 2201793
	Chiral metal nanocluster	Solution	10	0.0053	*Nano Res*. **2023**, 16, 7733
Nanocluster	Chiral metal nanocluster	Solution	20.8	0.0007	*Nat. Commun*. **2023**, 14, 4121
	**Chiral metal nanocluster**	**Solution**	**36.5**	**0.001**	**This work**

Reference

(1) Chu, Z.; Ye, Q.; Zhao, Y.; Ma, F.; Yin, Z.; Zhang, X.; You, J. Perovskite Light-Emitting Diodes with External Quantum Efficiency Exceeding 22% via Small-Molecule Passivation. *Adv. Mater.* **2021**, *33* (18), e2007169. DOI: 10.1002/adma.202007169.

(2) Sheldrick, G. M. SHELXT - integrated space-group and crystal-structure determination. *Acta Crystallogr. A Found. Adv.* **2015**, *71* (Pt 1), 3-8. DOI: 10.1107/S2053273314026370.

(3) Sheldrick, G. M. Crystal structure refinement with SHELXL. *Acta Crystallogr. C Struct. Chem.* **2015**, *71* (Pt 1), 3-8. DOI: 10.1107/S2053229614024218.

(4) Dolomanov, O. V.; Bourhis, L. J.; Gildea, R. J.; Howard, J. A. K.; Puschmann, H. OLEX2: a complete structure solution, refinement and analysis program. *J. Appl. Cryst.* **2009**, *42* (2), 339-341. DOI: doi:10.1107/S0021889808042726.

(5) Huang, R. W.; Song, X.; Chen, S.; Yin, J.; Maity, P.; Wang, J.; Shao, B.; Zhu, H.; Dong, C.; Yuan, P.; et al. Radioluminescent Cu-Au Metal Nanoclusters: Synthesis and Self-Assembly for Efficient X-ray Scintillation and Imaging. *J. Am. Chem. Soc.* **2023**, *145* (25), 13816-13827. DOI: 10.1021/jacs.3c02612.
